# Supplementary material for: Applying an osteopathic intervention to improve mild to moderate mental health symptoms: a mixed-methods feasibility study protocol
Source: BMJ Open. 2023 Jun 27;13(6):e071680. doi: 10.1136/bmjopen-2023-071680 (PMC10410888; doi:10.1136/bmjopen-2023-071680)
Supplement: Supplementary data [file bmjopen-2023-071680supp002.pdf]

Supplemental material 1.
Adverse events report form

Adverse Events Report Form		
**Practitioner ID:**		**Patient ID:**
**Date:**		**Location:**
**Description of adverse event:**		
**Actions taken:**<br>What?<br>When?<br>By whom?<br>Outcome?		
**Further actions needed?**		
