# Supplementary material for: Applying an osteopathic intervention to improve mild to moderate mental health symptoms: a mixed-methods feasibility study protocol
Source: BMJ Open. 2023 Jun 27;13(6):e071680. doi: 10.1136/bmjopen-2023-071680 (PMC10410888; doi:10.1136/bmjopen-2023-071680)
Supplement: Supplementary data [file bmjopen-2023-071680supp003.pdf]

Supplemental material 2.

Consent form

Swansea University
Prifysgol Abertawe

Participant Consent Form

Project title: **Measuring psychophysiological outcomes in a therapeutic touch approach.**

You must be age 18 or over to complete this study.

Name and Contact details of the principal researchers: Dr Josh Hope-Bell j.b.hope-bell@swansea.ac.uk and Dr Darren Edwards d.j.edwards@swansea.ac.uk.

This study is being conducted by Swansea University, Faculty of Medicine, Health and Life Sciences.

- I (the participant) consent to participate in the study
- I confirm that I have read and understand the information provided in relation to this study.
- I understand that my participation is voluntary. I understand that I am free to withdraw at any time during the study but once I have completed all phases of the study, withdrawal will not be possible because data will be completely anonymised.
- I understand what my role will be in this research, and all my questions have been answered to my satisfaction.
- I have been informed that the information I provide will be safeguarded.
- I am happy for the information I provide to be used (anonymously) in academic papers and other formal research outputs, however my name will not be published so anonymity is ensured.
- I agree to the researchers processing my personal data in accordance with the aims of the study described in the participant information.
- I am age 18 years or above.

If you agree with all statements above, click **Yes (I consent)**

If you disagree with any of the statements above, click **No (I do not consent)**
