# Supplementary material for: Applying an osteopathic intervention to improve mild to moderate mental health symptoms: a mixed-methods feasibility study protocol
Source: BMJ Open. 2023 Jun 27;13(6):e071680. doi: 10.1136/bmjopen-2023-071680 (PMC10410888; doi:10.1136/bmjopen-2023-071680)
Supplement: Author's manuscript [file bmjopen-2023-071680.draft_revisions.pdf]

BMJ Open is committed to open peer review. As part of this commitment we make the peer review history of every article we publish publicly available.

When an article is published we post the peer reviewers' comments and the authors' responses online. We also post the versions of the paper that were used during peer review. These are the versions that the peer review comments apply to.

The versions of the paper that follow are the versions that were submitted during the peer review process. They are not the versions of record or the final published versions. They should not be cited or distributed as the published version of this manuscript.

BMJ Open is an open access journal and the full, final, typeset and author-corrected version of record of the manuscript is available on our site with no access controls, subscription charges or pay-per-view fees (http://bmjopen.bmj.com).

If you have any questions on BMJ Open's open peer review process please email info.bmjopen@bmj.com

BMJ Open

Applying an osteopathic intervention to improve mild to moderate mental health symptoms: a mixed-methods feasibility study protocol

Journal:	*BMJ Open*
Manuscript ID	bmjopen-2023-071680
Article Type:	Protocol
Date Submitted by the Author:	10-Jan-2023

Complete List of Authors:	Hope-Bell, Josh; Swansea University, Department of Public Health<br>Draper-Rodi, Jerry; University College of Osteopathy, Research; National Council for Osteopathic Research,<br>Edwards, Darren; Swansea University, Department of Public Health

Keywords:	MENTAL HEALTH, Anxiety disorders < PSYCHIATRY, Depression & mood disorders < PSYCHIATRY

SCHOLARONE™ Manuscripts

Applying an osteopathic intervention to improve mild to moderate mental health symptoms: a mixed-methods feasibility study protocol

Josh Hope-Bell[1], Jerry Draper-Rodi[2,3] & Darren J. Edwards[1]

[1]Department of Public Health, Swansea University, UK

[2]National Council for Osteopathic Research, University College of Osteopathy, UK

[3] University College of Osteopathy, UK

For peer review only

Darren J. Edwards,

Department of Public Health,

Swansea University,

Swansea,

SA2 8PP,

UK

Email:d.j.edwards@swansea.ac.uk

Abstract

Introduction: Mental health services are stretched in the UK and are in need of support. One approach that could improve mental health symptoms is osteopathy. Research suggests that osteopathy influences psychophysiological factors, which could lead to improvements in mental health. The first objective of this protocol is to investigate the feasibility and acceptability of four osteopathic interventions. A secondary aim is to evaluate the interventions' effectiveness for improving psychophysiological and mental health outcomes.

Methods and analysis: This study will be an explanatory mixed-methods design (1). Participants will be 30 adults who have mild to moderate mental health symptoms and not experiencing any issues with pain. The feasibility and acceptability of the interventions will be the first primary outcome. Secondary outcomes will be physiological measures including heart rate variability (HRV), interoceptive accuracy (IAc) and blood pressure (BP). Psychological outcomes will also be measured by standardised questionnaires. These are being collected pre-and post-intervention. Additional outcomes will include recruitment rates and any adverse events that occur during the study. Participants will be randomised to one of four interventions. These are: (1) high-velocity and articulation techniques (HVAT), (2) soft-tissue massage (STM), (3) craniosacral therapy (CST), and (4) a combination of these three approaches. Participants will be interviewed about their experiences of the study and interventions. This will aid the assessment of the feasibility and acceptability of the study design.

Discussion: This study will assess the feasibility and acceptability of conducting osteopathic interventions for improving mental health outcomes. The results from this will help to inform the development of a future randomised controlled trial. The study will also produce original data which could provide preliminary evidence of whether osteopathic approaches are of benefit to individual's mental health in the form of effect sizes, even if they are pain-free.

For peer review only - http://bmjopen.bmj.com/site/about/guidelines.xhtml

Keywords: osteopathy, psychophysiology, mental health, feasibility study, randomised trial.

Strengths and limitations

- This study will investigate the utility of osteopathic techniques for improving mental health, in the absence of any existing pain
- The techniques being compared are based on previous literature and evidence
- Due to this being a feasibility study, only a small number of participants are being recruited which will lead to low statistical power of the results
- As the study is focused on comparing four interventions in a feasibility setting, there will be no control group comparison

Administrative information

This feasibility study protocol has followed the Standard Protocol Items: Recommendations for Intervention Trials (SPIRIT) guidelines (Chan et al., 2013).

1. Title

Applying an osteopathic intervention to improve mild to moderate mental health symptoms: a mixed-methods feasibility study protocol.

2. Trial registration

ClinicalTrials.gov Identifier: NCT05674071

3. Protocol Version

November 2022, v1.

4. Funding

The Osteopathic Foundation, grant award number: URNLG010.

5.1 Author details

Josh Hope-Bell & Darren J. Edwards, Department of Public Health, Swansea University.

Jerry Draper-Rodi, University College of Osteopathy and National Council for Osteopathic Research.

For peer review only

5.2 Name and contact information for the trial sponsor

The Osteopathic Foundation, 3 Park Terrace, Manor Road, Luton, Bedfordshire, LU1 3HN.

Tel: 01582 488 455. Email: enquiries@iosteopathy.org

5.3 Role of sponsor

The funders have no direct role in conducting this study. The funding is primarily being used to pay for the role of the research assistant on this project, held by JH-B.

Introduction

6.1 Background and rationale

In the UK, mental health problems such as anxiety and depression are an increasing burden within society. Recent estimates suggest that one in six people in the UK experience symptoms of depression or anxiety in any given week (2). For the individual, poor mental health can bring about problematic coping behaviours such as substance abuse and self-harm, leading to poor social relationships and in the worst cases; suicide (3). Mental health problems are commonly treated through psychotherapeutic means such as cognitive behavioural therapy, acceptance and commitment therapy, as well as relaxation techniques such as mindfulness practice and yoga (4). In addition, pharmacological solutions such as antidepressants and beta-blockers are used in treatment. These approaches have demonstrated effectiveness in many cases though they treat the symptoms and not the underlying causes (5,6).

With such a high demand being placed on the health services, such as these traditional forms of care, it can be difficult for many to receive treatment (7,8). It may therefore be important and helpful to consider innovative approaches that could support the demand for mental health services (9). Recently, it has been suggested that osteopathic interventions could be one such approach to support mental health services (10,11).

For peer review only

Osteopathy is an approach to health care that uses manual techniques to diagnose and treat patients (12). Osteopathy is an Allied Health Profession in England and osteopaths in the UK are regulated by statute (13). An osteopathic approach is patient-centred and focused on the patient's health rather than disease-centred. The practices are evidence-informed and scientific rigour forms an important part of treating patients and managing cases (14). Osteopaths use manual contact to identify and evaluate movement in all structural and functional aspects of the patient, identifying alterations of function and movement that impede health and addressing these. Osteopaths use a variety of techniques to manipulate joints, muscle, and tissue. All of the techniques used have an effect on the interplay between the nervous and musculoskeletal systems. Specific techniques include myofascial release, lymphatic drainage, high-velocity, low amplitude (HVLA), articulatory techniques and muscle energy techniques.

Previous studies have examined how osteopathy may influence psychophysiological factors. A number of these have examined the influence of osteopathic manipulative therapy (OMT) on heart rate variability (HRV), which is considered a potentially important indicator of physical and psychological wellbeing (15,16). Cerritelli et al. (17) found that two sessions of OMT significantly increased HRV in healthy adults, relative to a sham control group. Similarly, Arienti et al. (18) found that applying a single session fourth ventricle compression (CV4) technique significantly increased HRV, compared to a placebo intervention.

6.2 Choice of comparators

This study will be comparing four osteopathic interventions: (1) articulation and high-velocity thrust (HVT) techniques, (2) soft-tissue massage, (3) craniosacral techniques, and (4) a combination of all three techniques.

The choice of techniques in this study has been informed by a systematic review and meta-analysis by the authors into the impact of osteopathic interventions on

psychophysiological factors[1]. Articulation techniques were found to improve psychological outcomes (Castro-Sanchez et al., 2014; Espí-López et al., 2014), as well as autonomic nervous system indicators such as heart rate variability (HRV) and interoception (Cerritelli et al., 2020a). Similarly, interventions utilising HVT improved interoceptive accuracy and led to greater activation of brain areas associated with the interoceptive pathways (Cerritelli et al., 2020b). The studies that suggested articulation could improve psychological outcomes were conducted with patients with chronic pain and the study on HVT was with healthy participants. It will, therefore, be useful to understand whether articulation techniques and HVT could have positive psychological in the absence of pain and in the presence of mild mental health symptoms.

Next, soft-tissue massage techniques were chosen. Studies show that this approach has several positive psychological impacts for individuals with chronic pain (Baumgart et al., 2020) and pain-free patients who have mental health diagnoses (Rapaport et al., 2016; Sherman et al., 2010). Massage therapy has also been shown to have a preventative effect for general stress and wellbeing (Sharpe et al., 2007). Lastly, massage techniques have been shown to induce autonomic relaxation in healthy participants by increasing HRV (Seifert et al., 2018).

The third intervention will utilise craniosacral techniques. Three studies suggested that this approach induces autonomic relaxation by increasing HRV. One of these studies was conducted with patients with chronic pain (Castro-Sanchez et al., 2011) and two were carried out with healthy participants (Arienti et al., 2020; Edwards et al., 2018). It will therefore be useful to see whether any potential autonomic changes from craniosacral techniques translate

[1] This systematic review has been preregistered on OSF and the protocol is available via this link: https://osf.io/jrtpx/?view_only=63ffa916b76c4b95b4233d3cd812f12d

For peer review only - http://bmjopen.bmj.com/site/about/guidelines.xhtml

to psychological benefits. It will also be useful to examine the potential utility of these techniques with participants who have mild mental health symptoms.

The body areas that each intervention will focus on have also been informed by the aforementioned literature. The interventions will operate on a standardised protocol whereby body areas will be worked on in order by the practitioner. The body areas focussed on and the order they will be worked on will be described further in the specific procedures of each intervention.

7. Objectives

This study aims to investigate the feasibility and acceptability of four osteopathic interventions for adults with mild to moderate symptoms of mental health. A secondary aim is to evaluate the influence of these four interventions on physiological factors and their effectiveness for improving psychological outcomes. It is first hypothesised that the interventions will be feasible and acceptable to participants. It is also hypothesised that the interventions will induce psychophysiological relaxation by significantly increasing HRV and improving interoceptive accuracy. A final hypothesis is that the four interventions will lead to improvements on measures of mental health.

8. Trial design

This study will utilise an explanatory sequential mixed-methods approach. In this approach the quantitative aspect forms the first part of the study, followed by a qualitative aspect to help provide further explanation and depth (1). For the quantitative aspect, the study will utilise a parallel, randomised design with a 1:1 allocation to each of the four conditions. The qualitative aspect will be completed by interviewing the participants of the intervention and the practitioner delivering them.

Methods: Participants, interventions and outcomes.

9. Study setting

For peer review only

The study will take place at Swansea University in South Wales, UK. Participants will be recruited from both the student population at the university and the general public. Recruitment at the university will be conducted by advertising in communal spaces with posters. Additionally, social media will be used for recruitment by reaching out to mental health support groups and sharing an advertisement for the study on various social networks. The interventions are only taking place in one location and country: Wales, UK. The study will begin on the 14th of December 2022.

10. Eligibility Criteria

Eligibility criteria will include being over 18 years of age, experiencing mild to moderate symptoms of depression, stress or anxiety and able to read, write and speak English. Prospective participants will be excluded if they are experiencing acute or chronic pain, and/or if they have more severe mental health issues. The rationale for excluding participants with pain is that it may present a confounding variable. That is, if the osteopathic intervention alleviates any pain the participants are experiencing, this may lead to improvements in psychological symptoms. It would therefore not be clear whether osteopathy has a more *direct* influence on mental health outcomes.

11.1 Interventions

Participants will receive one of four interventions based on osteopathic techniques. All four interventions will consist of a single session lasting approximately 30 minutes. The interventions as follows: (1) articulation and high-velocity thrust (HVT) techniques, (2) soft-tissue massage, (3) craniosacral techniques, and (4) a combination of all three techniques. A summary of the intervention protocols can be found in Table 1.

Table 1.
Summary of the four intervention protocols and procedures.

For all	• 30-minute appointment	

For peer review only

	• Clinical findings, intervention, consent and adverse events (separate form to use if they do) recorded in participant individual form	**DURATION**
**Articulation / HVT group**	1. Observation + AROM (standing or sitting) + clinical examination for SD (sitting, prone or supine)<br>2. Techniques:<br>a. SD found: HVT to the area, unless contraindicated (info on BP/HA)<br>b. No SD found:<br>i. HVT TSp and ribs<br>ii. Articulation hips in extension	1. 10 min<br>2. 20 min
**Soft tissue group**	Full body, slow and superficial<br>1. Prone:<br>a. upper / mid / lower back<br>b. upper buttocks<br>c. hamstrings<br>d. calves<br>2. Supine:<br>a. Neck incl. suboccipital muscles<br>b. Shoulders<br>c. Pectoral muscles<br>d. Arms<br>e. Quadriceps<br>f. Feet	1. 15 min<br>2. 15 min
**Cranial group**	Looking for stiffness, asymmetry and tenderness on:<br>1. Sacrum<br>2. Head<br>Dysfunction found: myofascial release technique (10min/area); if no dysfunction found: functional techniques applied to each area (10min/area)<br>3. CV4	1. 10 min<br>2. 10 min<br>3. 10 min
**Combined group**	1. Observation + AROM + clinical exam for SD<br>2. HVT TSp<br>3. Soft tissue upper and lower back prone<br>4. CV4 and suboccipital release	1. 9 min<br>2. 7 min<br>3. 7 min<br>4. 7 min

Abbreviations: AROM: active range of movement; BP: blood pressure; CV4: Compression of the Fourth Ventricle technique; HA: headache; HVT: high velocity thrust techniques; SD: somatic dysfunction; TSp: thoracic spine

11.1.1 Articulation and high-velocity techniques

The articulation and high-velocity (AHVT) intervention will begin with an examination of the participant to search for somatic dysfunction (19). The AHVT

For peer review only

intervention will primarily be targeting all areas of the participant's spine. That is, the cervical, thoracic and lumbar areas, and also the sacroiliac joints. The practitioner will first observe the participant while standing, then will observe active range of movements in with the participant in standing and/or sitting positions. Then the practitioner will continue their examination searching first by light and then deeper palpation for signs associated with somatic dysfunction with the participant sitting down or lying prone or supine. This segment of the intervention will be allocated approximately 10 minutes.

If areas of the spine are found to have somatic dysfunction, then AHVT techniques will be applied to these areas. If no areas of somatic dysfunction are identified in the aforementioned spinal areas, then the practitioner will first focus on applying AHVT techniques to the thoracic spine and rib cage areas, followed by articulation techniques such as hip extension. The applying of techniques will be allocated approximately 20 minutes.

11.1.2 Soft-tissue massage techniques

The soft-tissue massage (STM) intervention will be a full-body massage. The participant will first be in the prone position and the practitioner will massage the upper, middle and lower areas of the back, the upper buttocks, then the hamstrings and calves. This will be approximately 15 minutes. The participant will then move into the supine position where they will receive massage on their neck, shoulders, pectoral muscles, arms, quadriceps and feet. This will also be allocated approximately 15 minutes. The literature suggests that slower techniques such as Swedish massage demonstrate effectiveness (20,21). There is also evidence that focussing on the upper layers of the skin has psychological benefits (22). These techniques will therefore be employed here.

11.1.3 Craniosacral techniques

This intervention will utilise craniosacral techniques (CST). This approach targets the cranial muscles and muscles around the central nervous system (23). The CST intervention will

For peer review only - http://bmjopen.bmj.com/site/about/guidelines.xhtml

begin with an examination for somatic dysfunction such as stiffness, asymmetry or tenderness. The body areas focussed on will be the head and sacrum which are body areas commonly associated with CST. If areas of dysfunction are identified, then the practitioner will perform myofascial release. If no areas of dysfunction in these areas are identified, the practitioner will first focus on the sacral region and then move on to other areas associated with CST. For sacral and cranial areas, approximately 10 minutes will be allocated each for 20 minutes total. The intervention will conclude with fourth ventricle compression (CV4). This technique is performed on the occipital bone. CV4 will be allocated approximately 10 minutes of the intervention.

11.1.4 Combination of techniques

This intervention will be a combination of all three techniques used in the other interventions (COMBO). The intervention will begin with an examination of the participant and checking active and passive range of movement. This examination will be allocated approximately 9 minutes. Using a combination of treatments the intervention will consist of: (1) high velocity techniques applied to the thoracic spine (approximately 7 minutes), (2) soft-tissue massage to the upper and lower back of the participant in prone (approximately 7 minutes), (3) CV4 and suboccipital muscles release (approximately 7 minutes). This intervention will therefore last approximately 30 minutes.

11.2 Modifications

In the interest of participant's safety, certain modifications may be made to the interventions if participants have body areas which are tender or if they present undiagnosed HBP. This is mostly relevant to the AVHT intervention and COMBO intervention which will have techniques that are of higher force. If a participant in the AVHT or COMBO interventions presents with HBP, neck pain or headaches during the intervention then the practitioner will not work on the cervical spine area and focus on the other spinal regions in the protocol.

For peer review only

11.3 Adherence

As the intervention only consists of one session, adherence is not necessarily applicable. Instead, a record will be kept of any participants who asked to end the intervention session early.

11.4 Concomitant care

Participants will be asked at pre-intervention if they are receiving any drug treatment for mental health (e.g., antidepressants), or psychotherapy (e.g., cognitive behavioural therapy). Participants will not be excluded on this basis, but these will be factored into the main statistical analysis as covariates.

12. Outcomes

12.1 Feasibility

The feasibility of the recruitment process will be determined by the number of people who respond to the advertisements and the number of people who are eligible/ineligible following the screening process. The feasibility of the measurement tools will first include whether participants have enough time to complete all measures. Feasibility of the questionnaires will also be assessed by any missing data. Additionally, the feasibility of the physiological measurements will be informed by the time taken to set up the equipment.

12.2 Acceptability

The acceptability of the study will be largely informed by the qualitative interview following the intervention. Participants will be asked about their experience of taking part, including what they preferred or did not prefer about the intervention and whether it has been useful to them. The practitioner will also be asked about their experience of delivering the interventions and for their feedback. Additionally, any adverse events occurring during the study will be logged using the Adverse Events Report Form (AERF; this can be found in Supplemental material 1.)

For peer review only - http://bmjopen.bmj.com/site/about/guidelines.xhtml

12.3 Psychological outcomes

These measures are intended to provide some initial data on the potential utility of the intervention for outcomes such as depression, anxiety and stress, psychological flexibility, and interoceptive awareness. They will be collected at pre- and post-intervention.

12.3.1 Depression, Anxiety and Stress Scale (DASS-21)

The DASS (24) is a self-report measure made up of 21 items with three subscales that measure depression, anxiety and stress. The DASS will also be used a screening tool to identify eligible participants in terms of the severity of mental health symptoms. Examples of items include "I couldn't seem to experience any positive feeling at all" for the depression scale, "I felt I was close to panic" for the anxiety scale, and "I found myself getting agitated" for the stress scale. These are then rated on a four-point Likert scale ranging from 0 (never) to 3 (almost always). Higher scores indicate higher levels of depression, anxiety and stress. The subscales have good internal reliability as measured by Cronbach's alpha coefficients (α), which are 0.88 for depression, 0.82 for anxiety and 0.90 for stress, as well as 0.93 the total score (25).

12.3.2 International Positive and Negative Affect Schedule- Short-Form (PANAS-SF)

The PANAS-SF (26) is a short-form version of the PANAS and uses 10 items to measure two subscales of positive and negative affect (PA and NA). Participants are asked to what extent they have felt certain states or emotions, such as "inspired" for the PA scale and "upset" for the NA scale. These are then rated on a five-point Likert scale ranging from 1 (very slightly or not at all) to 5 (extremely). Higher scores indicate higher levels of PA and NA. Both the PA and NA subscales have good internal reliability with both having a Cronbach's α of 0.84 (26).

12.3.3 Acceptance and Action Questionnaire-II (AAQ-II)

For peer review only

For peer review only - http://bmjopen.bmj.com/site/about/guidelines.xhtml

The AAQ-II (27) is a self-report measure made up of 7 items that measures psychological inflexibility or as it also referred to, experiential avoidance. Items include a list of statements such as "I'm afraid of my feelings" and "worries get in the way of my success". These items are then rated on a seven-point Likert scale from 1 (never true) to 7 (always true). Scores are then totalled with higher scores indicating greater levels of psychological inflexibility and experiential avoidance. The AAQ-II has good internal reliability with a Cronbach's α of 0.84 (27).

12.3.4 Self as Context Scale (SACS)

The SACS (28) uses 10 items to measure self-as-context, one of the acceptance components of psychological flexibility. Self-as-context can be described as a transcendent sense of self, where the individual is able to distance their "noticing self" from internal thoughts and feelings. The SACS has two subscales, (1) centering e.g., "when I am upset, I am able to find a place of calm within myself", and (2) transcending e.g., "As I look back upon my life so far, I have a sense that part of me has been there for all of it". Items are then rated on a seven-point Likert scale from 1 (strongly disagree) to 7 (strongly agree). Higher scores on the subscales indicate higher levels of centering, transcending and a higher total score indicates greater levels of self-as-context. The SACS has good internal reliability with Cronbach's α of 0.81 for centering, 0.78 for transcending and 0.81 for overall SACS score (28).

12.3.5 Multidimensional Assessment of Interoceptive Awareness Version 2 (MAIA-2)

The MAIA-2 (29) is a 37-item self-report measure of interoceptive awareness. The MAIA-2 uses eight subscales which are: (1) noticing e.g., "when I am tense, I notice where the tension is located in my body", (2) not-distracting e.g., "I distract myself from sensations of discomfort", (3) not-worrying e.g., "when I feel physical pain, I become upset", (4) attention regulation (e.g., "I can pay attention to my breath without being distracted by things happening around me"), (5) emotional awareness e.g., "I notice how my body changes when

For peer review only - http://bmjopen.bmj.com/site/about/guidelines.xhtml

I am angry", (6) self-regulation e.g., "when I feel overwhelmed I can find a calm place inside", (7) body listening e.g., "I listen for information from my body about my emotional state, and (8) trusting e.g., "I trust my body sensations". The items are rated on a six-point Likert scale ranging from 0 (never) to 5 (always). The scales have good internal reliability with the Cronbach's alpha coefficients for the scales being: 0.64 for noticing, 0.74 for not-distracting, 0.67 for not-worrying, 0.83 for attention regulation, 0.79 for emotional awareness, 0.79 for self-regulation, 0.80 for body listening, and 0.83 for trust (29).

12.4 Physiological outcomes

12.4.1 Heart rate variability (HRV)

HRV will be measured using a medical-grade Holter electrocardiogram (ECG) monitor. Measurements will be taken at two timepoints, pre- and post-intervention. A time-domain signal measure will be calculated using root mean square of successive interval differences (RMSSD). Frequency-domain measurements will be calculated by using low frequency power, high frequency power and low frequency to high frequency ratio (LF/HF).

12.4.2 Interoceptive accuracy (IAc)

Participants will perform a heartbeat detection task as measure of IAc. This is conducted in the form of the heartbeat perception task which is performed according to the Mental Tracking Method (30) using intervals of 30, 35, 40, and 45s that are separated by 30s resting periods. During each trial R–R intervals are recorded, and participants are asked to silently count their heartbeats without the use of an exteroceptive aid (such as taking one's pulse). At the end of each period participants verbally report the number of counted heartbeats. The participants are not informed about the length of the counting phases nor about the quality of their performance. Interoceptive sensibility will also be measured through participant's subjective assessments about how accurately they perceived heartbeats (31). These measures will be completed pre- and post-intervention.

For peer review only - http://bmjopen.bmj.com/site/about/guidelines.xhtml

12.4.3 Blood pressure (BP)

BP will be measured at pre- and post-intervention. This will be carried out in line with the National Institute for Health and Care Excellence (NICE) recommendations. That is, BP will be collected in a room which is quiet, relaxed, and temperate, whilst the participant will be quiet and seated, and their arm outstretched and supported, using an appropriate cuff size for the person's arm (32). This outstretching of the arm will allow the practitioner to assess any undiagnosed high blood-pressure (HBP). If the participant has HBP it can make some of the osteopathic techniques less safe, so it is important to establish this. HBP will be determined according to the NICE recommendation of BP results that are 140/90 mmHg and over (32). In addition to participant safety, measuring BP will provide data of any impact the intervention might have on this physiological indicator.

12.5 Additional outcomes

Demographic information will also be collected from participants relating to their gender, age and ethnic background. Although participants will have been screened for chronic pain, they will be asked whether they are currently or have recently been experiencing any neck pain or headaches. This is to inform the clinician about any problematic body areas, which may therefore be avoided in the intervention. The participants should be presenting as pain-free due to the initial screening process, but this is still a necessary safety measure. Participants will not be excluded if they present neck pain or headaches. However, if a number of participants indicate they are experiencing neck pain or headaches, this may be explored in the analysis as a covariate.

Participants will also be asked about whether they are currently receiving any mental health treatment. They will be asked whether they are currently taking any antidepressants or other related prescribed medication for mental health issues. Participants will also be asked whether they have recently attended or are currently attending any form of talking therapy or

For peer review only - http://bmjopen.bmj.com/site/about/guidelines.xhtml

other psychotherapy. Participant's prescription medication or psychotherapy status will not exclude them from the study. However, this will again be entered as a covariate if a number of participants report that they are receiving these psychological treatments.

Lastly will be the noting of any adverse events that occur during the intervention or study period. These will be filled out by the practitioner using the AERF and collected by the researcher if occurring during the intervention. If participants contact the researcher after the intervention regarding an adverse event, then this will be logged by the researcher.

12.6 Qualitative outcomes

Participants will be interviewed about their experience of the intervention via telephone approximately one week after they have completed the study. The interviews will be semi-structured and follow a pre-defined schedule (see Table 2). The interview will be centred around the acceptability of the intervention, but also aspects of the study. To this end the interview will ask questions about motivations for taking part and expectations, how informed they felt before taking part, their experience of completing the questionnaires and having physiological measures taken, and their experience of the intervention itself. Some questions will also ask about participants how they have felt since the intervention. Participants will then be given a chance to provide any other feedback or thoughts on taking part in the study. The audio from the interviews will be recorded and then transcribed. Analysis of the data will be conducted using reflexive thematic analysis (Braun & Clarke, 2021). This involves initially familiarising oneself with the transcripts and then coding the data. Codes are then collated into themes. From here, themes are refined and categorised into main themes, midlevel themes and subthemes. Themes will then be discussed in terms of their strength. That is an indication will be provided of whether themes were common across many participants' accounts, or only mentioned by a few. It is hoped that by employing

For peer review only

qualitative methods, a richer account of the acceptability of the study and intervention to participants can be obtained.

The practitioner will also be interviewed about their experience of *delivering* the intervention. This interview will also be thematically analysed, and the resulting themes explored.

Table 2.
Interview schedule for qualitative interviews.

**Information and consent**	1. Were there any parts of the information sheet that were difficult to understand?<br>2. Did any part of your participation feel unexpected, based on what you were told in the information sheet?
**Motivations for participating**	3. What motivated you to participate in the study?<br>4. What did you know about osteopathy before taking part?
**Outcome measures- questionnaires**	5. Were there any questions or words on the questionnaires that were difficult to understand?<br>6. What was your experience like filling out the questionnaires?
**Outcome measures- physiological**	7. What was your experience of having an ECG and blood pressure taken?<br>8. What was your experience of doing a heartbeat detection task?
**Intervention**	9. Did you feel that the practitioner adequately explained procedures to you?<br>10. What could have gone better during the session?<br>11. Did you take anything useful away from the session or learn anything new?<br>12. How likely are you to visit an osteopath again or seek similar treatments after this?
**Closing points**	13. What else could you tell us about your experience of taking part in this study?

13. Participant timeline

See Table 3 for the participant timeline.

For peer review only - http://bmjopen.bmj.com/site/about/guidelines.xhtml

Table 3.
Participant timeline

		T_{-1}	T_0	T_1	T_2	T_3	F_1
Activity/ Assessment	Approx. time to complete	Pre-study Screening/ consent	Pre-study randomisation	Pre-intervention	Intervention	Post-intervention tests	Follow-up 1-week
Informed consent	5 mins	X					
Screening with DASS	5 mins	X					
Randomisation	15 mins		X				
Baseline assessment-questionnaires	15 mins			X			
Baseline assessment-physiological	15 mins			X			
Intervention	30 mins				X		
Post-intervention questionnaires	15 mins					X	
Post-intervention physiological	15 mins					X	
Telephone interview	30 mins						X

Abbreviations: T: Timepoint; F: Follow-up; DASS: Depression Anxiety Stress Scale.

14. Sample Size

The study will aim to recruit 30 participants. This number of participants is generally deemed sufficient for feasibility studies (33). In terms of practical constraints, it also represents the maximum number that can be accommodated with the resources available.

15. Recruitment

Participants will contact the research team if they are interested in taking part. They will then be given an information sheet to read and consent form to sign. Following this they will complete the Depression, Anxiety, Stress Scale (DASS) to complete to determine their eligibility regarding mental health symptoms. If eligible they will be invited to take part in the intervention. If they display severe mental health symptoms, they will not be invited to take part further and signposted to the relevant mental health services and charities.

Methods: Assignment of interventions

For peer review only

16. Allocation

16.1 Sequence generation

Participants will be randomly assigned to one of the four conditions using a computerised random number generator. Permuted block randomisation will be used to ensure that equal numbers of participants are in each condition. The block sizes will not be disclosed to help ensure concealment and prevent any potential prediction of group allocation. This will be conducted by the PI of the study DJE, whilst the researcher (out-come assessor) JH-B is blinded to this randomisation process.

16.2 Concealment mechanism

Allocation concealment will be ensured using sequentially numbered, sealed opaque envelopes which contain the group assignment. The PI of the study DJE, will carry out the allocation concealment, and ensure that the researcher (out-come assessor) JH-B is blinded to the intervention allocation.

16.3 Implementation

All participants who provide informed consent and who meet the eligibility criteria will be randomised into a study condition (as described in section 16.1). The randomisation DJE will not be directly involved in the recruitment or data collection, and instead the researcher JHB (outcome assessor) will conduct the recruitment. The list of random numbers that correspond to group allocation will not be revealed to outcome assessor JHB involved in data collection or recruitment. The sealed envelopes will contain a randomisation number and corresponding intervention identity code for the allocation of participants into intervention group. The osteopathic practitioner will then be able to open the envelope and determine which intervention is to be delivered on the day the study is conducted.

17.1 Blinding (masking)

For peer review only

For peer review only - http://bmjopen.bmj.com/site/about/guidelines.xhtml

The outcome assessor JHB will be blind to participants' group allocation. After pre-intervention psychometric and psychophysiological measures (see section 12.3 and 12.4 respectively) have been completed, the outcome assessor will leave the room (to ensure blinding) and the intervention will begin, conducted by the osteopath. Participants will not be blinded to study intervention, as the osteopathic practitioner will need to explain study and intervention procedures, in line with the osteopathic practice standards (14) and ethical consent. The practitioner will not be blinded to the intervention type (as they need to know what intervention to deliver) but will be blinded to the study outcomes. The outcome assessor will also be conducting the data analysis, and the random numbers corresponding to each group will only be revealed when this analysis has been completed. To ensure participants do not disclose the condition they were allocated to, they will be asked not to communicate directly to the outcome assessor about the intervention they received. The study will therefore be single-blinded, where the outcome assessor is blind to intervention allocation, and the osteopathic practitioner will not be blind (hence single-blind).

17.2 Emergency unblinding

As the practitioner is not blinded, no emergency unblinding procedures are deemed necessary.

Methods: Data management and analysis

18. Data management

All data will be entered electronically at the university where the data is being collected and kept in a password-protected folder, where only the outcome assessor (JHB) will have access to for the duration of the study. The electronic data will be kept confidential, and participant's names will not be linked to their dataset. For the longer term, electronic datasets will be kept indefinitely in the interest of transparency to fulfil any requests for the original data and maintained on the Open Science Framework (OSF).

For peer review only

19. Statistical Methods

19.1 Outcomes

Statistical analysis will be conducted using SPSS (IBM, 2022). Mean and standard deviation will be reported for demographic data that includes gender, age, and ethnicity. For the main analysis, data will first be examined for normality using the Shapiro-Wilk test. If data is skewed, logarithmic transformation will be used, otherwise analysis will continue without any transformation. HRV data will be pre-processed, inspected for any potential artifacts, where these will be removed if identified. RMSSD will be calculated on the pre-processed artifact removed data using Kubios version 3.5[2] via Matlab version R2021a. Interoceptive accuracy (IAc) will then be calculated using the formula: IAc = 1/4 Σ [1 − (|recorded heartbeats − counted heartbeats|/recorded heartbeats)]. The psychometrics will be totalled according to the relevant questionnaire instructions and subscales.

The main analysis will comprise of seven separate mixed design two (pre- and post-intervention) by four (AVHT, STM, CST, combination[3]) analysis of covariance (ANCOVA) models. This will comprise of five separate ANCOVAs for the five psychometrics (DASS, PANAS-SF, AAQ, SACS, MAIA) and another two ANCOVAs for the physiological measures of IAc and HRV (as measured by RMSSD and LF/HF ratio). Covariates will consist of: (1) whether participants are currently receiving psychotherapy (yes or no) and (2) whether participants are receiving pharmacological treatment (yes or no). Significant models will be examined further using post hoc Bonferroni tests.

19.2 Additional Analyses

---
[2] https://www.kubios.com/
[3] Please see: Interventions section 11.1 for full details of these interventions.

For peer review only

Exploratory correlational analyses will also be conducted to examine relationships between changes from pre- to post-intervention on the various measures (e.g., relationship between change from pre-post HRV RMSSD and pre-post DASS scores).

19.3 Analysis population and missing data

The study will operate on an intention-to-treat basis. All participants randomised and with pre-intervention data will be included in the final analysis. Any participants with missing data will be included in the analysis using the multiple imputation feature of SPSS.

Methods: Monitoring

20. Data monitoring

As this study is taking place over a short duration as a feasibility study and not a full RCT, no formal committee for data monitoring is required.

21. Harms

The osteopathic practitioner will inform the participants about the general potential common adverse effects of osteopathy namely some stiffness and soreness in the days following the intervention, and rare adverse events including tissue damage, in line with informed consent processes. The osteopathic practitioner will record any adverse effects one the day the intervention is received (that occur during or immediately after the intervention) in the Adverse Events report form (see supplemental material 1). Participants will also be advised to contact the PI DJE if they have any concerns or adverse events following the intervention in subsequent days after the intervention was received. If such events are reported, these will again be reported by DJE in the Adverse Events report. Any adverse events or harms that are ranked highly on severity will be reported to the ethical committee.

22. Auditing

For peer review only

As the study is taking place over a short duration and only at one site, no formal auditing processes are deemed necessary, though PI will have regular team meetings to ensure the study is following the research protocol at all times.

Methods: Patient and public involvement statement

Key stakeholders were consulted and involved at a very early stage of the research process. The Patient Experience and Evaluation in Research (Patient Experience and Evaluation in Research (PEER): https://www.swansea.ac.uk/humanandhealthsciences/research-at-the-college-of-human-and-health/patientexperienceandevaluationinresearchpeergroup/) group in the College of Human and Health Sciences at Swansea University were consulted. This group represented members of the public, students, and staff members, several of whom reported that they had experienced depression, anxiety or stress at some point in their lives and emphasised the need for innovative approaches of the delivery of mental health support. The feasibility design was explained to them, and they gave positive feedback about the nature of the preliminary research plan.

Ethics and dissemination

23. Research ethics approval

The protocol for this feasibility study has received ethical approval from the Department of Psychology Ethics Committee at Swansea University, ethical review reference number: 2022-5603-4810.

24. Protocol amendments

Any deviations from the protocol that could impact the conduct or bias of the study will be clearly outlined and justified in the final written report. Version control of the protocol using identifiers and dates, along with a list of amendments will be clearly listed. This will enable tracking of the history of amendments and identification of the most recent protocol version.

25. Consent

For peer review only - http://bmjopen.bmj.com/site/about/guidelines.xhtml

Participants will scan a QR code on recruitment posters or click a link via email/social media adverts that will take them to the study's information sheet. The information sheet emphasises that participation is voluntary and that they can withdraw from the study at any stage, without needing to provide a reason. If they have any questions or concerns at this stage, they are encouraged on the information sheet to contact the research team. If they are willing to proceed, they will complete an online consent form (see Supplemental material 2).

26. Ancillary research

The data collected in this study will not be used for any other ancillary research.

27. Confidentiality

Participants will be assigned a coded ID number to maintain confidentiality. Any records of personal identifiers such as informed consent forms will be stored separately to data with ID numbers. To limit data access to the minimum number of individuals, only the researcher JHB will have access to the data for analysis.

28. Declaration of interests

This projected is being funded by the Osteopathic Foundation (OF). The OF has no direct input into the study. The individual authors have no direct conflicts of interest to declare.

29. Access to data

Only the researcher JHB will have access to the dataset during the study period. Upon completion, the collected data will be deidentified and made available on the Open Science Framework (OSF). Similarly, the SPSS statistical syntax code used will be made available on OSF.

30. Ancillary and post-trial care

Participants will be fully debriefed once they have completed the study. The contact details of the research team will be provided should participants have any concerns. As the participants will be presenting with mild to moderate mental health symptoms, the debrief form will

For peer review only - http://bmjopen.bmj.com/site/about/guidelines.xhtml

encourage participants to seek support services such as mental health charities or their GP if their psychological condition deteriorates at any time.

31. Dissemination policy

31.1 Trial results

Following the completion of the study, it is anticipated to take around 2-3 months to compile the final results ready for publication to an appropriate peer-reviewed journal. The study's results may also be used as part of presentations at any relevant conferences.

31.2 Authorship

The authors of this protocol will also be the authors on the final report. All authors have made substantive contributions to the design of the study. Additionally, all authors will have made substantive contributions to the interpretation of the data collected and the writing of the final report.

31.3 Reproducible research

This protocol will be available to researchers via open access publication. The dataset collected will be deidentified and made available on OSF. Similarly, the statistical syntax code used will be made available on OSF. These will be made available no later than 1 year upon completion of data collection.

Acknowledgements

We would like to thank Stephen Hartshorn for his contribution in delivering the osteopathic interventions.

Contributorship statement

JHB wrote the first draft, then all authors subsequently revised additional drafts. All authors have made substantive contributions to the concept, design and writing of this study.

Competing interests

For peer review only

There are no competing interests. This projected is funded by the Osteopathic Foundation (OF). The OF has no direct input into any aspects of this study. The individual authors have no direct conflicts of interest to declare.

Funding

This research has been funded by The Osteopathic Foundation, grant award number: URNLG010.

For peer review only

References

1. Creswell JW, Plano-Clark VickiL. Designing and Conducting Mixed Methods Research. 3rd ed. Los Angeles: SAGE Publishing; 2017.
2. McManus S, Bebbington P, Jenkins R, Brugha T. Mental health and wellbeing in England: Adult Psychiatric Morbidity Survey 2014. Leeds; 2016.
3. WHO. Suicide worldwide in 2019: global health estimates. Geneva; 2021.
4. Edwards DJ. The textbook of health and social care. Washington, D.C: SAGE Publications Ltd; 2020. (Core textbook).
5. Cipriani A, Furukawa TA, Salanti G, Chaimani A, Atkinson LZ, Ogawa Y, et al. Comparative efficacy and acceptability of 21 antidepressant drugs for the acute treatment of adults with major depressive disorder: a systematic review and network meta-analysis. The Lancet. 2018 Apr 7; 391(10128):1357–66. Available from: http://www.thelancet.com/article/S0140673617328027/fulltext
6. Munder T, Flückiger C, Leichsenring F, Abbass AA, Hilsenroth MJ, Luyten P, et al. Is psychotherapy effective? A re-analysis of treatments for depression. Epidemiol Psychiatr Sci. 2019 Jun 1; 28(3):268–74. Available from: https://www.cambridge.org/core/journals/epidemiology-and-psychiatric-sciences/article/is-psychotherapy-effective-a-reanalysis-of-treatments-for-depression/5D8EC85B6FA35B5CEE124381F18E51B9
7. Wang PS, Berglund PA, Olfson M, Kessler RC. Delays in Initial Treatment Contact after First Onset of a Mental Disorder. Health Serv Res. 2004 Apr 1; 39(2):393–416. Available from: https://onlinelibrary.wiley.com/doi/full/10.1111/j.1475-6773.2004.00234.x

For peer review only - http://bmjopen.bmj.com/site/about/guidelines.xhtml

8. Patel V, Maj M, Flisher AJ, de Silva MJ, Koschorke M, Prince M, et al. Reducing the treatment gap for mental disorders: a WPA survey. World Psychiatry. 2010 Oct 1; 9(3):169–76. Available from: https://onlinelibrary.wiley.com/doi/full/10.1002/j.2051-5545.2010.tb00305.x

9. Edwards DJ, Kemp AH. A novel ACT-based video game to support mental health through embedded learning: a mixed-methods feasibility study protocol. BMJ Open. 2020 Nov 1;10(11):e041667. Available from: https://bmjopen.bmj.com/content/10/11/e041667

10. Bohlen L, Shaw R, Cerritelli F, Esteves JE. Osteopathy and Mental Health: An Embodied, Predictive, and Interoceptive Framework. Front Psychol. 2021 Oct 27;12:4989.

11. Edwards DJ, Toutt C. An evaluation of osteopathic treatment on psychological outcomes with patients suffering from chronic pain: A prospective observational cohort study collected through a health and well-being academy. Health Psychol Open. 2018 Jan 1; 5(1). Available from: https://pubmed.ncbi.nlm.nih.gov/29780605/

12. Vaucher P, Macdonald RJD, Carnes D. The role of osteopathy in the Swiss primary health care system: a practice review. BMJ Open. 2021 Oct 1; 8(8):e023770. Available from: https://bmjopen.bmj.com/content/8/8/e023770

13. General Osteopathic Council. General Osteopathic Council. https://www.osteopathy.org.uk/home/. 2022.

14. General Osteopathic Council. Osteopathic Practice Standards. London; 2019.

15. Kemp AH, Quintana DS, Felmingham KL, Matthews S, Jelinek HF. Depression, Comorbid Anxiety Disorders, and Heart Rate Variability in Physically Healthy, Unmedicated Patients: Implications for Cardiovascular Risk. PLoS One. 2012 Feb 15; 7(2):e30777. Available from: https://journals.plos.org/plosone/article?id=10.1371/journal.pone.0030777

16. Pinna T, Edwards DJ. A Systematic Review of Associations Between Interoception, Vagal Tone, and Emotional Regulation: Potential Applications for Mental Health, Wellbeing, Psychological Flexibility, and Chronic Conditions. Front Psychol. 2020 Aug 5;11:1792.

17. Cerritelli F, Cardone D, Pirino A, Merla A, Scoppa F. Does Osteopathic Manipulative Treatment Induce Autonomic Changes in Healthy Participants? A Thermal Imaging Study. Front Neurosci. 2020 Aug 18;14:887.

For peer review only

18. Arienti C, Farinola F, Ratti S, Daccò S, Fasulo L. Variations of HRV and skin conductance reveal the influence of CV4 and Rib Raising techniques on autonomic balance: A randomized controlled clinical trial. J Bodyw Mov Ther. 2020 Oct 1; 24(4):395–401. Available from: http://www.bodyworkmovementtherapies.com/article/S1360859220301303/fulltext

19. Fryer G. Somatic dysfunction: An osteopathic conundrum. International Journal of Osteopathic Medicine. 2016 Dec 1;22:52–63.

20. Rapaport MH, Schettler P, Larson ER, Edwards SA, Dunlop BW, Rakofsky JJ, et al. Acute Swedish Massage Monotherapy Successfully Remediates Symptoms of Generalized Anxiety Disorder: A Proof-of-Concept, Randomized Controlled Study. J Clin Psychiatry. 2016 Jul 1; 77(7):e883–91. Available from: https://pubmed.ncbi.nlm.nih.gov/27464321/

21. Sharpe PA, Williams HG, Granner ML, Hussey JR. A randomised study of the effects of massage therapy compared to guided relaxation on well-being and stress perception among older adults. Complement Ther Med. 2007 Sep 1;15(3):157–63.

22. Baumgart SBE, Baumbach Kraft A, Lorenz J. Effect of Psycho-Regulatory Massage Therapy on Pain and Depression in Women with Chronic and/or Somatoform Back Pain: A Randomized Controlled Trial. Brain Sci. 2020 Oct 1;10(10):1–13. Available from: https://pubmed.ncbi.nlm.nih.gov/33053728/

23. Gabutti M, Draper-Rodi J. Osteopathic decapitation: Why do we consider the head differently from the rest of the body? New perspectives for an evidence-informed osteopathic approach to the head. International Journal of Osteopathic Medicine. 2014 Dec 1;17(4):256–62.

24. Lovibond PF, Lovibond SH. The structure of negative emotional states: Comparison of the Depression Anxiety Stress Scales (DASS) with the Beck Depression and Anxiety Inventories. Behaviour Research and Therapy. 1995 Mar 1;33(3):335–43.

25. Henry JD, Crawford JR. The short-form version of the Depression Anxiety Stress Scales (DASS-21): Construct validity and normative data in a large non-clinical sample. British Journal of Clinical Psychology. 2005 Jun 1; 44(2):227–39. Available from: https://onlinelibrary.wiley.com/doi/full/10.1348/014466505X29657

26. Thompson ER. Development and Validation of an Internationally Reliable Short-Form of the Positive and Negative Affect Schedule (PANAS). J Cross Cult Psychol. 2007 Mar 1;38(2):227–42. Available from: https://doi.org/10.1177/0022022106297301

For peer review only

27. Bond FW, Hayes SC, Baer RA, Carpenter KM, Guenole N, Orcutt HK, et al. Preliminary Psychometric Properties of the Acceptance and Action Questionnaire–II: A Revised Measure of Psychological Inflexibility and Experiential Avoidance. Behav Ther. 2011 Dec 1;42(4):676–88.
28. Zettle RD, Gird SR, Webster BK, Carrasquillo-Richardson N, Swails JA, Burdsal CA. The Self-as-Context Scale: Development and preliminary psychometric properties. J Contextual Behav Sci. 2018 Oct 1;10:64–74.
29. Mehling WE, Acree M, Stewart A, Silas J, Jones A. The Multidimensional Assessment of Interoceptive Awareness, Version 2 (MAIA-2). PLoS One. 2018 Dec 4;13(12):e0208034-. Available from: https://doi.org/10.1371/journal.pone.0208034
30. Schandry R. Heart Beat Perception and Emotional Experience. Psychophysiology. 1981 Jul 1;18(4):483–8. Available from: https://onlinelibrary.wiley.com/doi/full/10.1111/j.1469-8986.1981.tb02486.x
31. Garfinkel SN, Seth AK, Barrett AB, Suzuki K, Critchley HD. Knowing your own heart: Distinguishing interoceptive accuracy from interoceptive awareness. Biol Psychol. 2015;104:65–74. Available from: https://www.sciencedirect.com/science/article/pii/S0301051114002294
32. NICE. Hypertension in adults: diagnosis and management. London; 2022.
33. Lancaster GA, Dodd S, Williamson PR. Design and analysis of pilot studies: recommendations for good practice. J Eval Clin Pract. 2004 May;10(2):307–12. Available from: https://pubmed.ncbi.nlm.nih.gov/15189396/

For peer review only

Supplemental material 1.
Adverse events report form

**Adverse Events Report Form**		
**Practitioner ID:**		**Patient ID:**
**Date:**		**Location:**
**Description of adverse event:**		
**Actions taken:** What? When? By whom? Outcome?		
**Further actions needed?**		

For peer review only

STANDARD PROTOCOL ITEMS: RECOMMENDATIONS FOR INTERVENTIONAL TRIALS

SPIRIT 2013 Checklist: Recommended items to address in a clinical trial protocol and related documents*

Section/item	Item No	Description	Addressed on page number
**Administrative information**			
Title	1	Descriptive title identifying the study design, population, interventions, and, if applicable, trial acronym	Page 3, Section 1.
Trial registration	2a	Trial identifier and registry name. If not yet registered, name of intended registry	Page 3, Section 2.
	2b	All items from the World Health Organization Trial Registration Data Set	N/A
Protocol version	3	Date and version identifier	Page 3, Section 3.
Funding	4	Sources and types of financial, material, and other support	Page 3, Section 4.
Roles and responsibilities	5a	Names, affiliations, and roles of protocol contributors	Pages 3-4, Section 5.1
	5b	Name and contact information for the trial sponsor	Page 4, Section 5.2
	5c	Role of study sponsor and funders, if any, in study design; collection, management, analysis, and interpretation of data; writing of the report; and the decision to submit the report for publication, including whether they will have ultimate authority over any of these activities	Page 4, Section 5.3

For peer review only

For peer review only - http://bmjopen.bmj.com/site/about/guidelines.xhtml

	5d	Composition, roles, and responsibilities of the coordinating centre, steering committee, endpoint adjudication committee, data management team, and other individuals or groups overseeing the trial, if applicable (see Item 21a for data monitoring committee)	N/A
**Introduction**			
Background and rationale	6a	Description of research question and justification for undertaking the trial, including summary of relevant studies (published and unpublished) examining benefits and harms for each intervention	Pages 4-5, Section 6.1
	6b	Explanation for choice of comparators	Pages 5-7, Section 6.2
Objectives	7	Specific objectives or hypotheses	Page 7, Section 7
Trial design	8	Description of trial design including type of trial (eg, parallel group, crossover, factorial, single group), allocation ratio, and framework (eg, superiority, equivalence, noninferiority, exploratory)	Page 7, Section 8
**Methods: Participants, interventions, and outcomes**			
Study setting	9	Description of study settings (eg, community clinic, academic hospital) and list of countries where data will be collected. Reference to where list of study sites can be obtained	Pages 7-8, Section 9
Eligibility criteria	10	Inclusion and exclusion criteria for participants. If applicable, eligibility criteria for study centres and individuals who will perform the interventions (eg, surgeons, psychotherapists)	Page 8, Section 10
Interventions	11a	Interventions for each group with sufficient detail to allow replication, including how and when they will be administered	Pages 8-11, Section 11.1
	11b	Criteria for discontinuing or modifying allocated interventions for a given trial participant (eg, drug dose change in response to harms, participant request, or improving/worsening disease)	Page 11, Section 11.2
	11c	Strategies to improve adherence to intervention protocols, and any procedures for monitoring adherence (eg, drug tablet return, laboratory tests)	Page 11-12, Section 11.3

For peer review only - http://bmjopen.bmj.com/site/about/guidelines.xhtml

	11d	Relevant concomitant care and interventions that are permitted or prohibited during the trial	Page 12, Section 11.4
Outcomes	12	Primary, secondary, and other outcomes, including the specific measurement variable (eg, systolic blood pressure), analysis metric (eg, change from baseline, final value, time to event), method of aggregation (eg, median, proportion), and time point for each outcome. Explanation of the clinical relevance of chosen efficacy and harm outcomes is strongly recommended	Pages 12-18, Section 12
Participant timeline	13	Time schedule of enrolment, interventions (including any run-ins and washouts), assessments, and visits for participants. A schematic diagram is highly recommended (see Figure)	Pages 18-19, Section 13 (Table 3)
Sample size	14	Estimated number of participants needed to achieve study objectives and how it was determined, including clinical and statistical assumptions supporting any sample size calculations	Page 19, Section 14
Recruitment	15	Strategies for achieving adequate participant enrolment to reach target sample size	Page 19, Section 15.

Methods: Assignment of interventions (for controlled trials)

Allocation:

Sequence generation	16a	Method of generating the allocation sequence (eg, computer-generated random numbers), and list of any factors for stratification. To reduce predictability of a random sequence, details of any planned restriction (eg, blocking) should be provided in a separate document that is unavailable to those who enrol participants or assign interventions	Page 20, Section 16.1
Allocation concealment mechanism	16b	Mechanism of implementing the allocation sequence (eg, central telephone; sequentially numbered, opaque, sealed envelopes), describing any steps to conceal the sequence until interventions are assigned	Page 20, Section 16.2
Implementation	16c	Who will generate the allocation sequence, who will enrol participants, and who will assign participants to interventions	Page 20, Section 16.3
Blinding (masking)	17a	Who will be blinded after assignment to interventions (eg, trial participants, care providers, outcome assessors, data analysts), and how	Pages 20-21, Section 17.1

For peer review only - http://bmjopen.bmj.com/site/about/guidelines.xhtml

	17b	If blinded, circumstances under which unblinding is permissible, and procedure for revealing a participant's allocated intervention during the trial	Page 21, Section 17.2.
**Methods: Data collection, management, and analysis**			
Data collection methods	18a	Plans for assessment and collection of outcome, baseline, and other trial data, including any related processes to promote data quality (eg, duplicate measurements, training of assessors) and a description of study instruments (eg, questionnaires, laboratory tests) along with their reliability and validity, if known. Reference to where data collection forms can be found, if not in the protocol	Pages 12-18, Section 12.
	18b	Plans to promote participant retention and complete follow-up, including list of any outcome data to be collected for participants who discontinue or deviate from intervention protocols	N/A as data only collected pre-post.
Data management	19	Plans for data entry, coding, security, and storage, including any related processes to promote data quality (eg, double data entry; range checks for data values). Reference to where details of data management procedures can be found, if not in the protocol	Page 21, Section 18.
Statistical methods	20a	Statistical methods for analysing primary and secondary outcomes. Reference to where other details of the statistical analysis plan can be found, if not in the protocol	Page 21-22, Section 19.1
	20b	Methods for any additional analyses (eg, subgroup and adjusted analyses)	Page 22, Section 19.2
	20c	Definition of analysis population relating to protocol non-adherence (eg, as randomised analysis), and any statistical methods to handle missing data (eg, multiple imputation)	Page 22, Section 19.3
**Methods: Monitoring**			
Data monitoring	21a	Composition of data monitoring committee (DMC); summary of its role and reporting structure; statement of whether it is independent from the sponsor and competing interests; and reference to where further details about its charter can be found, if not in the protocol. Alternatively, an explanation of why a DMC is not needed	Page 23, Section 20.
	21b	Description of any interim analyses and stopping guidelines, including who will have access to these interim results and make the final decision to terminate the trial	N/A

Harms	22	Plans for collecting, assessing, reporting, and managing solicited and spontaneously reported adverse events and other unintended effects of trial interventions or trial conduct	Page 23, Section 21.
Auditing	23	Frequency and procedures for auditing trial conduct, if any, and whether the process will be independent from investigators and the sponsor	Page 23, Section 22.
**Ethics and dissemination**			
Research ethics approval	24	Plans for seeking research ethics committee/institutional review board (REC/IRB) approval	Page 24, Section 23.
Protocol amendments	25	Plans for communicating important protocol modifications (eg, changes to eligibility criteria, outcomes, analyses) to relevant parties (eg, investigators, REC/IRBs, trial participants, trial registries, journals, regulators)	Page 24, Section 24
Consent or assent	26a	Who will obtain informed consent or assent from potential trial participants or authorised surrogates, and how (see Item 32)	Page 24, Section 25
	26b	Additional consent provisions for collection and use of participant data and biological specimens in ancillary studies, if applicable	N/A
Confidentiality	27	How personal information about potential and enrolled participants will be collected, shared, and maintained in order to protect confidentiality before, during, and after the trial	Pages 24-25, Section 27.
Declaration of interests	28	Financial and other competing interests for principal investigators for the overall trial and each study site	Page 25, Section 28.
Access to data	29	Statement of who will have access to the final trial dataset, and disclosure of contractual agreements that limit such access for investigators	Page 25, Section 29.
Ancillary and post-trial care	30	Provisions, if any, for ancillary and post-trial care, and for compensation to those who suffer harm from trial participation	Page 25, Section 30.
Dissemination policy	31a	Plans for investigators and sponsor to communicate trial results to participants, healthcare professionals, the public, and other relevant groups (eg, via publication, reporting in results databases, or other data sharing arrangements), including any publication restrictions	Page 25, Section 31.1

For peer review only

	31b	Authorship eligibility guidelines and any intended use of professional writers	Page 25, Section 31.2
	31c	Plans, if any, for granting public access to the full protocol, participant-level dataset, and statistical code	Page 26, Section 31.3
**Appendices**			
Informed consent materials	32	Model consent form and other related documentation given to participants and authorised surrogates	Supplemental material 2
Biological specimens	33	Plans for collection, laboratory evaluation, and storage of biological specimens for genetic or molecular analysis in the current trial and for future use in ancillary studies, if applicable	N/A

*It is strongly recommended that this checklist be read in conjunction with the SPIRIT 2013 Explanation & Elaboration for important clarification on the items. Amendments to the protocol should be tracked and dated. The SPIRIT checklist is copyrighted by the SPIRIT Group under the Creative Commons "Attribution-NonCommercial-NoDerivs 3.0 Unported" license.

For peer review only

BMJ Open

Applying an osteopathic intervention to improve mild to moderate mental health symptoms: a mixed-methods feasibility study protocol

Journal:	*BMJ Open*
Manuscript ID	bmjopen-2023-071680.R1
Article Type:	Protocol
Date Submitted by the Author:	23-May-2023
Complete List of Authors:	Hope-Bell, Josh; Swansea University, Department of Public Health<br>Draper-Rodi, Jerry; University College of Osteopathy, Research; National Council for Osteopathic Research,<br>Edwards, Darren; Swansea University, Department of Public Health
**Primary Subject Heading**:	Complementary medicine
Secondary Subject Heading:	Mental health
Keywords:	MENTAL HEALTH, Anxiety disorders < PSYCHIATRY, Depression & mood disorders < PSYCHIATRY

SCHOLARONE™
Manuscripts

Applying an osteopathic intervention to improve mild to moderate mental health symptoms: a mixed-methods feasibility study protocol

Josh Hope-Bell[1], Jerry Draper-Rodi[2,3] & Darren J. Edwards[1]

[1]Department of Public Health, Swansea University, UK

[2]National Council for Osteopathic Research, University College of Osteopathy, UK

[3] University College of Osteopathy, UK

For peer review only

Darren J. Edwards,

Department of Public Health,

Swansea University,

Swansea,

SA2 8PP,

UK

Email:d.j.edwards@swansea.ac.uk

For peer review only - http://bmjopen.bmj.com/site/about/guidelines.xhtml

Abstract

Introduction: Mental health services are stretched in the UK and are in need of support. One approach that could improve mental health symptoms is osteopathy. Research suggests that osteopathy influences psychophysiological factors, which could lead to improvements in mental health. The first objective of this protocol is to investigate the feasibility and acceptability of four osteopathic interventions. A secondary aim is to evaluate the interventions' effectiveness for improving psychophysiological and mental health outcomes.

Methods and analysis: This study will be an explanatory mixed-methods design (1). Participants will be 30 adults who have mild to moderate mental health symptoms and not experiencing any issues with pain. The feasibility and acceptability of the interventions will be the primary outcomes. Secondary outcomes will be physiological measures including heart rate variability (HRV), interoceptive accuracy (IAc), and blood pressure (BP). Psychological outcomes, collected pre-and post-intervention, will also be measured by five standardised questionnaires, which include: (1) the Depression, Anxiety and Stress Scale; (2) the International Positive and Negative Affect Schedule- Short-Form; (3) Acceptance and Action Questionnaire-II ; (4) the Self as Context Scale; (5) and the Multidimensional Assessment of Interoceptive Awareness Version 2. Participants will be randomised to one of four intervention groups and receive a single intervention treatment session. These intervention groups are: (1) high-velocity and articulation techniques (HVAT), (2) soft-tissue massage (STM), (3) craniosacral therapy (CST), and (4) a combination of these three approaches. Mixed design two (pre- and post-intervention) by the four interventions analysis of covariance (ANCOVA) models will be used to analyse the quantitative data for each quantitative measure. Participants will also be interviewed about their experiences of the study and interventions and a thematic analysis will be used to analyse this qualitative data. This will aid the assessment of the feasibility and acceptability of the study design.

For peer review only - http://bmjopen.bmj.com/site/about/guidelines.xhtml

Discussion: This study will assess the feasibility and acceptability of conducting osteopathic interventions for improving mental health outcomes. The results from this will help to inform the development of a future randomised controlled trial. The study will also produce original data which could provide preliminary evidence of whether osteopathic approaches are of benefit to individual's mental health in the form of effect sizes, even if they are pain-free.

Trial registration: ClinicalTrials.gov Identifier: NCT05674071

Keywords: osteopathy, psychophysiology, mental health, feasibility study, randomised trial.

Strengths and limitations

- This study will investigate the utility of osteopathic techniques for improving mental health, in the absence of any existing pain
- The techniques being compared are based on previous literature and evidence
- Due to this being a feasibility study, only a small number of participants are being recruited which will lead to low statistical power of the results
- As the study is focused on comparing four interventions in a feasibility setting, there will be no control group comparison

Administrative information

This feasibility study protocol has followed the Standard Protocol Items: Recommendations for Intervention Trials (SPIRIT) guidelines (see Supplemental material 1) (1).

1. Title

Applying an osteopathic intervention to improve mild to moderate mental health symptoms: a mixed-methods feasibility study protocol.

2. Trial registration

ClinicalTrials.gov Identifier: NCT05674071

3. Protocol Version

November 2022, v1.

For peer review only

4. Funding

The Osteopathic Foundation, grant award number: UR5NLG010.

5.1 Author details

Josh Hope-Bell & Darren J. Edwards, Department of Public Health, Swansea University.

Jerry Draper-Rodi, University College of Osteopathy and National Council for Osteopathic Research.

5.2 Name and contact information for the trial sponsor

The Osteopathic Foundation, 3 Park Terrace, Manor Road, Luton, Bedfordshire, LU1 3HN. Tel: 01582 488 455. Email: enquiries@iosteopathy.org

5.3 Role of sponsor

The funders have no direct role in conducting this study. The funding is primarily being used to pay for the role of the research assistant on this project, held by JH-B.

Introduction

6.1 Background and rationale

In the UK, mental health problems such as anxiety and depression are an increasing burden within society. Recent estimates suggest that one in six people in the UK experience symptoms of depression or anxiety in any given week (2). For the individual, poor mental health can bring about problematic coping behaviours such as substance abuse and self-harm, leading to poor social relationships and in the worst cases; suicide (3). Mental health problems are commonly treated through psychotherapeutic means such as cognitive behavioural therapy, acceptance and commitment therapy (ACT), as well as relaxation techniques such as mindfulness practice and yoga (4). In addition, pharmacological solutions such as antidepressants and beta-blockers are used in treatment. These approaches have demonstrated effectiveness in many cases though they treat the symptoms and not the underlying causes (5, 6).

For peer review only

With such a high demand being placed on the health services, such as these traditional forms of care, it can be difficult for many to receive treatment (7, 8). It may therefore be important and helpful to consider innovative approaches that could support the demand for mental health services (9). Recently, it has been suggested that osteopathic interventions could be one such approach to support mental health services (10, 11).

Osteopathy is an approach to health care that uses manual techniques to diagnose and treat patients (12). Osteopathy is an Allied Health Profession in England and osteopaths in the UK are regulated by statute (13). An osteopathic approach is patient-centred and focused on the patient's health rather than disease-centred. The practices are evidence-informed and scientific rigour forms an important part of treating patients and managing cases (14). Osteopaths use manual contact to identify and evaluate movement in all structural and functional aspects of the patient, identifying alterations of function and movement that impede health and addressing these. Osteopaths use a variety of techniques to manipulate joints, muscles, and tissue. All of the techniques used have an effect on the interplay between the nervous and musculoskeletal systems (15-17). Specific techniques include myofascial release, lymphatic drainage, high-velocity, low amplitude (HVLA), articulatory techniques, and muscle energy techniques.

The rationale for linking mental health with physiological mechanisms comes as previous studies have examined how osteopathy may influence psychophysiological factors. A number of these have examined the influence of osteopathic manipulative therapy (OMT) on heart rate variability (HRV), which is considered a potentially important indicator of physical and psychological wellbeing (18, 19). Cerritelli et al. (20) found that two sessions of OMT significantly increased HRV in healthy adults, relative to a sham control group. Similarly, Arienti et al. (21) found that applying a single session of fourth ventricle compression (CV4) significantly increased HRV, compared to a placebo intervention. So,

For peer review only - http://bmjopen.bmj.com/site/about/guidelines.xhtml

there seems to be some clear evidence that influencing physiological mechanisms through osteopathy are highly relevant for mental health improvement. Indeed, studies have found that osteopathic interventions have led to improvements in mental health outcomes such as anxiety (22), depression (23), and stress (24). However, few studies have sought to examine the effects of osteopathy on both physiological *and* psychological outcomes.

This feasibility protocol will, therefore, explore both the potential psychological and psychophysiological changes that occur after osteopathic treatment, in a small group of individuals who suffer from mild to moderate forms of anxiety, stress, and depression.

6.2 Choice of comparators

This study will be comparing four osteopathic interventions: (1) articulation and high-velocity thrust (HVT) techniques, (2) soft-tissue massage, (3) craniosacral techniques, and (4) a combination of all three techniques.

The choice of techniques in this study has been informed by a systematic review and meta-analysis by the authors into the impact of osteopathic interventions on psychophysiological factors[1]. This review included randomised controlled trials of manual interventions and their effects on factors including mental health outcomes and physiological indicators such as HRV and interoception. Articulation techniques were found to improve psychological outcomes (25, 26), as well as autonomic nervous system indicators such as heart rate variability (HRV) and interoception (27). Similarly, interventions utilising HVT improved interoceptive accuracy and led to greater activation of brain areas associated with the interoceptive pathways (28). The studies that suggested articulation could improve psychological outcomes were conducted with chronic pain patients and the study on HVT with healthy participants. It will, therefore, be useful to understand whether articulation

[1] This systematic review has been preregistered on OSF and the protocol is available via this link: https://osf.io/jrtpx/?view_only=63ffa916b76c4b95b4233d3cd812f12d

For peer review only

techniques and HVT could have positive psychological effects in the absence of pain and in the presence of mild mental health symptoms.

Next, soft-tissue massage techniques were chosen. Studies show that this approach has several positive psychological impacts for individuals with chronic pain (23) and pain-free patients who have mental health diagnoses (22, 29). Massage therapy has also been shown to have a preventative effect for general stress and wellbeing (24). Lastly, massage techniques have been shown to induce autonomic relaxation in healthy participants by increasing HRV (30).

The third intervention will utilise craniosacral techniques. Three studies suggested that this approach induces autonomic relaxation by increasing HRV. One of these studies was conducted with patients with chronic pain (31) and two were carried out with healthy participants (21, 32). It will therefore be useful to see whether any potential autonomic changes from craniosacral techniques translate to psychological benefits. It will also be useful to examine the potential utility of these techniques with participants who have mild mental health symptoms.

The body areas that each intervention will focus on have also been informed by the aforementioned literature. The interventions will operate on a standardised protocol whereby body areas will be worked on in order by the practitioner. The body areas focussed on and the order they will be worked on will be described further in the specific procedures of each intervention.

7. Objectives

This study aims to investigate the feasibility and acceptability of four osteopathic interventions for adults with mild to moderate mental health symptomatology. A secondary aim is to evaluate the influence of these four interventions on physiological factors including HRV and interoception. The study will aim to evaluate their effectiveness in improving

For peer review only

psychological outcomes including depression, stress, anxiety, negative affect, and psychological flexibility. It is first hypothesised that the interventions will be feasible and acceptable to participants. It is also hypothesised that the interventions will induce psychophysiological relaxation by significantly increasing HRV and improving interoceptive accuracy. No specific predictions are made about blood pressure as data on this is only being collected to check that we are within safe blood pressure bounds, i.e., for participant safety. A final hypothesis is that the four interventions will lead to similar improvements in depression, anxiety, stress, and negative affect.

8. Trial design

This is a feasibility study, which will utilise an explanatory sequential mixed-methods approach. In this approach the quantitative aspect forms the first part of the study, followed by a qualitative aspect to help provide further explanation and depth (33). For the quantitative aspect, the study will utilise a parallel, randomised design with an equal proportion of participants allocated to each of the four conditions. The qualitative aspect will be completed by interviewing the participants of the intervention and the practitioner delivering them.

Methods: Participants, interventions, and outcomes.

9. Study setting

The study will take place at Swansea University in South Wales, UK with participants being recruited from both the student population at the university and the general public. The interventions will only take place in one location and country: Wales, UK. The study began on December 20th, 2022, and the study will be completed by August 1st, 2023.

10. Eligibility Criteria

Eligibility criteria will include being over 18 years of age, experiencing mild to moderate symptoms of depression, stress, or anxiety, and being able to read, write and speak English. Prospective participants will be excluded if they are experiencing acute or chronic

For peer review only - http://bmjopen.bmj.com/site/about/guidelines.xhtml

pain, and/or if they have no psychological symptoms or more severe mental health issues. The rationale for excluding participants with pain is that it may present a confounding variable. That is, if the osteopathic intervention alleviates any pain the participants are experiencing, this may lead to improvements in psychological symptoms. It would therefore not be clear whether osteopathy has a more *direct* influence on mental health outcomes. Screening for mild to moderate psychological symptoms will be conducted using the Depression, Anxiety, Stress Scale (DASS).

11.1 Interventions

Participants will receive one of four interventions based on osteopathic techniques. All four interventions will consist of a single session lasting approximately 30 minutes. The interventions are being delivered by two male osteopaths, one with 17 years of practice experience and one with 3 years of practice experience. The interventions will be as follows: (1) articulation and high-velocity thrust (HVT) techniques, (2) soft-tissue massage, (3) craniosacral techniques, and (4) a combination of all three techniques. A summary of the intervention protocols can be found in Table 1.

Table 1.
Summary of the four intervention protocols and procedures.

For all	• 30-minute appointment<br>• Clinical findings, intervention, consent, and adverse events (separate form to use if they do) recorded in participant individual form	**DURATION**
**Articulation / HVT group**	1. Observation + AROM (standing or sitting) + clinical examination for SD (sitting, prone, or supine)<br>2. Techniques:<br>a. SD found: HVT to the area unless contraindicated (info on BP/HA)<br>b. No SD found:<br>i. HVT TSp and ribs<br>ii. Articulation of hips in extension	1. 10 min<br>2. 20 min
**Soft tissue group**	Full body, slow and superficial<br>1. Prone:<br>a. upper / mid / lower back	1. 15 min

For peer review only

	b. upper buttocks<br>c. hamstrings<br>d. calves<br>2. Supine:<br>a. Neck incl. suboccipital muscles<br>b. Shoulders<br>c. Pectoral muscles<br>d. Arms<br>e. Quadriceps<br>f. Feet	 <br><br>2. 15 min
**Cranial group**	Looking for stiffness, asymmetry, and tenderness on:<br>1. Sacrum<br>2. Head<br>Dysfunction found: myofascial release technique (10min/area); if no dysfunction found: functional techniques applied to each area (10min/area)<br>3. CV4	 1. 10 min<br>2. 10 min<br><br><br><br>3. 10 min
**Combined group**	1. Observation + AROM + clinical exam for SD<br>2. HVT TSp<br>3. Soft tissue upper and lower back prone<br>4. CV4 and suboccipital release	1. 9 min<br>2. 7 min<br>3. 7 min<br>4. 7 min

Abbreviations: AROM: active range of movement; BP: blood pressure; CV4: Compression of the Fourth Ventricle technique; HA: headache; HVT: high velocity thrust techniques; SD: somatic dysfunction; TSp: thoracic spine

11.1.1 Articulation and high-velocity techniques

The articulation and high-velocity (AHVT) intervention will begin with an examination of the participant to search for somatic dysfunction (34). The AHVT intervention will primarily be targeting all areas of the participant's spine. That is, the cervical, thoracic, and lumbar areas, and also the sacroiliac joints. The practitioner will first observe the participant while standing, then will observe active range of movements with the participant in standing and/or sitting positions. Then the practitioner will continue their examination searching first by light and then deeper palpation for signs associated with somatic dysfunction with the participant sitting down or lying prone or supine. This segment of the intervention will be allocated approximately 10 minutes.

For peer review only

If areas of the spine are found to have somatic dysfunction, then AHVT techniques will be applied to these areas. If no areas of somatic dysfunction are identified in the aforementioned spinal areas, then the practitioner will first focus on applying AHVT techniques to the thoracic spine and rib cage areas, followed by articulation techniques such as hip extension. The application of techniques will be allocated approximately 20 minutes.

11.1.2 Soft-tissue massage techniques

The soft-tissue massage (STM) intervention will be a full-body massage. The participant will first be in the prone position and the practitioner will massage the upper, middle, and lower areas of the back, the upper buttocks, then the hamstrings and calves. This will be approximately 15 minutes. The participant will then move into the supine position where they will receive massage on their neck, shoulders, pectoral muscles, arms, quadriceps, and feet. This will also be allocated approximately 15 minutes. The literature suggests that slower techniques such as Swedish massage demonstrate effectiveness (24, 29). There is also evidence that focusing on the upper layers of the skin has psychological benefits (23). These techniques will therefore be employed here.

11.1.3 Craniosacral techniques

This intervention will utilise craniosacral techniques (CST). This approach targets the cranial muscles and muscles around the central nervous system (35). The CST intervention will begin with an examination for somatic dysfunction such as stiffness, asymmetry, or tenderness. The body areas focussed on will be the soft tissue around the head and sacrum areas which are body areas commonly associated with CST. If areas of dysfunction are identified, then the practitioner will perform myofascial release. If no areas of dysfunction in these areas are identified, the practitioner will first focus on the sacral region and then move on to other areas associated with CST. For sacral and cranial areas, approximately 10 minutes will be allocated each for 20 minutes total. The intervention will conclude with fourth

For peer review only - http://bmjopen.bmj.com/site/about/guidelines.xhtml

ventricle compression (CV4). This technique is performed on the occipital bone. CV4 will be allocated approximately 10 minutes of the intervention.

11.1.4 Combination of techniques

This intervention will be a combination of all three techniques used in the other interventions (COMBO). The intervention will begin with an examination of the participant and checking active and passive range of movement. This examination will be allocated approximately 9 minutes. Using a combination of treatments, the intervention will consist of: (1) high-velocity techniques applied to the thoracic spine (approximately 7 minutes), (2) soft-tissue massage to the upper and lower back of the participant in prone (approximately 7 minutes), (3) CV4 and suboccipital muscles release (approximately 7 minutes). This intervention will therefore last approximately 30 minutes.

11.2 Modifications

In the interest of participant's safety, certain modifications may be made to the interventions if participants have body areas that are tender or if they present undiagnosed high blood pressure (HBP). This is mostly relevant to the AVHT intervention and COMBO intervention which will have techniques that are of higher force. If a participant in the AVHT or COMBO interventions presents with HBP, neck pain, or headaches during the intervention then the practitioner will not work on the cervical spine area and focus on the other spinal regions in the protocol. The justification is that HVT techniques may increase the risk of arterial damage in individuals with HBP (36, 37).

11.3 Adherence

As the intervention only consists of one session, adherence is not necessarily applicable. Instead, a record will be kept of any participants who asked to end the intervention session early.

11.4 Concomitant care

For peer review only - http://bmjopen.bmj.com/site/about/guidelines.xhtml

Participants will be asked at pre-intervention if they are receiving any drug treatment for mental health (e.g., antidepressants), or psychotherapy (e.g., cognitive behavioural therapy). Participants will not be excluded on this basis, but these will be factored into the main statistical analysis as covariates.

12. Outcomes

The primary outcomes are the feasibility and acceptability outcomes (12.1 and 12.2), whilst the secondary outcomes are the psychological outcome measures (12.3.1 to 12.3.5) and the psychophysiological measures (12.4.1 to 12.4.3).

12.1 Feasibility

The feasibility of the recruitment process will be determined by the number of people who respond to the advertisements and the number of people who are eligible/ineligible following the screening process. Specifically, recruitment will be considered feasible if more than 100 people respond and if at least half of the responders are eligible following screening. The feasibility of the measurement tools will first include whether participants have enough time to complete all measures. The feasibility of the questionnaires will also be assessed by any missing data. Additionally, the feasibility of the physiological measurements will be informed by the time taken to set up the equipment. The measures will therefore be considered feasible if they can all be completed in the allotted time (approximately 40 minutes).

12.2 Acceptability

The acceptability of the study will be largely informed by the qualitative interview following the intervention.

Participants will be interviewed about their experience of the intervention via telephone approximately one week after they have completed the study. The interviews will be semi-structured and follow a pre-defined schedule (see Table 2). The interview will be centred

For peer review only

around the acceptability of the intervention, but also aspects of the study. To this end, the interview will ask questions about motivations for taking part and expectations, how informed they felt before taking part, their experience of completing the questionnaires and having physiological measures taken, and their experience of the intervention itself. Some questions will also ask participants how they have felt since the intervention. Participants will then be given a chance to provide any other feedback or thoughts on taking part in the study. The audio from the interviews will be recorded and then transcribed.

Analysis of the data will be conducted using reflexive thematic analysis (38). This involves initially familiarising oneself with the transcripts and then coding the data. Codes are then collated into themes. From here, themes are refined and categorised into main themes, midlevel themes, and subthemes. Themes will then be discussed in terms of their strength. That is an indication will be provided of whether themes were common across many participants' accounts, or only mentioned by a few. It is hoped that by employing qualitative methods, a richer account of the acceptability of the study and intervention to participants can be obtained.

The practitioners will also be interviewed about their experience of *delivering* the intervention. This interview will also be thematically analysed, and the resulting themes explored.

Table 2.
Interview schedule for qualitative interviews.

**Information and consent**	1. Were there any parts of the information sheet that were difficult to understand?<br>2. Did any part of your participation feel unexpected, based on what you were told in the information sheet?
**Motivations for participating**	3. What motivated you to participate in the study?<br>4. What did you know about osteopathy before taking part?

For peer review only

**Outcome measures-questionnaires**	5. Were there any questions or words on the questionnaires that were difficult to understand?<br>6. What was your experience like filling out the questionnaires?
**Outcome measures-physiological**	7. What was your experience of having an ECG and blood pressure taken?<br>8. What was your experience of doing a heartbeat detection task?
**Intervention**	9. Did you feel that the practitioner adequately explained the procedures to you?<br>10. What could have gone better during the session?<br>11. Did you take anything useful away from the session or learn anything new?<br>12. How likely are you to visit an osteopath again or seek similar treatments after this?
**Closing points**	13. What else could you tell us about your experience of taking part in this study?

Additionally, any adverse events occurring during the study will be logged using the Adverse Events Report Form (AERF; this can be found in Supplemental material 2).

12.3 Psychological outcomes

These measures are intended to provide some initial data on the potential utility of the intervention for outcomes such as depression, anxiety and stress, psychological flexibility, and interoceptive awareness. They will be collected during pre- and post-intervention and any changes will be analysed.

12.3.1 Mental health

Depression, Anxiety, and Stress Scale (DASS-21)

The DASS (39) is a self-report measure made up of 21 items with three subscales that measure depression, anxiety, and stress. The DASS will also be used as a screening tool to

For peer review only - http://bmjopen.bmj.com/site/about/guidelines.xhtml

identify eligible participants in terms of the severity of mental health symptoms. Examples of items include “I couldn’t seem to experience any positive feeling at all” for the depression scale, “I felt I was close to panic” for the anxiety scale, and “I found myself getting agitated” for the stress scale. These are then rated on a four-point Likert scale ranging from 0 (never) to 3 (almost always). Higher scores indicate higher levels of depression, anxiety, and stress. The subscales have good internal reliability as measured by Cronbach’s alpha coefficients (α), which are 0.88 for depression, 0.82 for anxiety, and 0.90 for stress, as well as 0.93 for the total score (40).

International Positive and Negative Affect Schedule- Short-Form (PANAS-SF)

The PANAS-SF (41) is a short-form version of the PANAS and uses 10 items to measure two subscales of positive and negative affect (PA and NA). Participants are asked to what extent they have felt certain states or emotions, such as “inspired” for the PA scale and “upset” for the NA scale. These are then rated on a five-point Likert scale ranging from 1 (very slightly or not at all) to 5 (extremely). Higher scores indicate higher levels of PA and NA. Both the PA and NA subscales have good internal reliability with both having a Cronbach’s α of 0.84 (41).

12.3.2 Psychological flexibility

Acceptance and Action Questionnaire-II (AAQ-II)

The AAQ-II (42) is a self-report measure made up of 7 items that measures psychological inflexibility or as it is also referred to, experiential avoidance. Items include a list of statements such as “I’m afraid of my feelings” and “worries get in the way of my success”. These items are then rated on a seven-point Likert scale from 1 (never true) to 7 (always true). Scores are then totalled with higher scores indicating greater levels of psychological inflexibility and experiential avoidance. The AAQ-II has good internal reliability with a Cronbach’s α of 0.84 (42).

For peer review only - http://bmjopen.bmj.com/site/about/guidelines.xhtml
For peer review only

Self as Context Scale (SACS)

The SACS (43) uses 10 items to measure self-as-context, one of the acceptance components of psychological flexibility. Self-as-context can be described as a transcendent sense of self, where the individual is able to distance their "noticing self" from internal thoughts and feelings. The SACS has two subscales, (1) centering e.g., "when I am upset, I am able to find a place of calm within myself", and (2) transcending e.g., "As I look back upon my life so far, I have a sense that part of me has been there for all of it". Items are then rated on a seven-point Likert scale from 1 (strongly disagree) to 7 (strongly agree). Higher scores on the subscales indicate higher levels of centering, and transcending and a higher total score indicates greater levels of self-as-context. The SACS has good internal reliability with Cronbach's α of 0.81 for centering, 0.78 for transcending, and 0.81 for overall SACS score (43).

12.3.3 Interoceptive awareness

Multidimensional Assessment of Interoceptive Awareness Version 2 (MAIA-2)

The MAIA-2 (44) is a 37-item self-report measure of interoceptive awareness. The MAIA-2 uses eight subscales which are: (1) noticing e.g., "when I am tense, I notice where the tension is located in my body", (2) not-distracting e.g., "I distract myself from sensations of discomfort", (3) not-worrying e.g., "when I feel physical pain, I become upset", (4) attention regulation e.g., "I can pay attention to my breath without being distracted by things happening around me", (5) emotional awareness e.g., "I notice how my body changes when I am angry", (6) self-regulation e.g., "when I feel overwhelmed I can find a calm place inside", (7) body listening e.g., "I listen for information from my body about my emotional state, and (8) trusting e.g., "I trust my body sensations". The items are rated on a six-point Likert scale ranging from 0 (never) to 5 (always). The scales have good internal reliability with the Cronbach's alpha coefficients for the scales being: 0.64 for noticing, 0.74 for not-distracting,

For peer review only - http://bmjopen.bmj.com/site/about/guidelines.xhtml

0.67 for not-worrying, 0.83 for attention regulation, 0.79 for emotional awareness, 0.79 for self-regulation, 0.80 for body listening, and 0.83 for trust (44).

12.4 Physiological outcomes

These measures will provide initial data on how the intervention impacts psychophysiological factors. These measures will be collected during pre- and post-intervention and any changes analysed. The physiological measures are all being conducted in the same environment.

12.4.1 Heart rate variability (HRV)

HRV will be measured using a medical-grade Holter electrocardiogram (ECG) monitor. Measurements will be taken at two time points, pre-and post-intervention. Participants will lie in a supine position while the ECG monitor records for at least 5 minutes. Participants will be asked in advance to refrain from consuming any caffeine, alcohol, or nicotine on the day of the study, to minimise interference with the ECG. A time-domain signal measure will be calculated using the root mean square of successive interval differences (RMSSD). Frequency-domain measurements will also be calculated by using low-frequency power, high-frequency power, and low-frequency to high-frequency ratio (LF/HF). This measure will be analysed in conjunction with the recent literature that suggest it is a measure of primarily the parasympathetic system (45).

12.4.2 Interoceptive accuracy (IAc)

Participants will perform a heartbeat detection task as a measure of IAc. This is conducted in the form of the heartbeat perception task which is performed according to the Mental Tracking Method (46) using intervals of 30, 35, 40, and 45s that are separated by 30s resting periods. During each trial R–R intervals are recorded, and participants are asked to silently count their heartbeats without the use of an exteroceptive aid (such as taking one's pulse). At the end of each period, participants verbally report the number of counted heartbeats. The participants will not be informed about the length of the counting phases nor the quality of

For peer review only

their performance. Interoceptive sensibility will also be measured through participants' subjective assessments about how accurately they perceived heartbeats (47). These measures will be completed pre-and post-intervention.

12.4.3 Blood pressure (BP)

BP will be measured at pre-and post-intervention. This will be carried out in line with the National Institute for Health and Care Excellence (NICE) recommendations. That is, BP will be collected in a room that is quiet, relaxed, and temperate, whilst the participant will be quiet and seated, and their arm outstretched and supported, using an appropriate cuff size for the person's arm (48). This outstretching of the arm will allow the practitioner to assess any undiagnosed HBP. If the participant has HBP it can make some of the osteopathic techniques less safe (36, 37), so it is important to establish this. HBP will be determined according to the NICE recommendation of BP results that are 140/90 mmHg and over (48). In addition to participant safety, measuring BP will provide data on any impact the intervention might have on this physiological indicator.

12.5 Additional outcomes

Demographic information will also be collected from participants relating to their gender, age, and ethnic background. Although participants will have been screened for chronic pain, they will be asked whether they are currently or have recently been experiencing any neck pain or headaches. This is to inform the clinician about any problematic body areas, which may therefore be avoided in the intervention. The participants should be presenting as pain-free due to the initial screening process, but this is still a necessary safety measure. Participants will be excluded from the analysis if they present with neck pain or headaches.

Participants will also be asked whether they are currently receiving any mental health treatment. They will be asked whether they are currently taking any antidepressants or other related prescribed medication for mental health issues. Participants will also be asked

For peer review only

whether they have recently attended or are currently attending any form of talking therapy or other psychotherapy. Participants' prescription medication or psychotherapy status will not exclude them from the study. However, this will again be entered as a covariate if several participants report that they are receiving these psychological treatments.

Lastly will be the noting of any adverse events that occur during the intervention or study period. These will be filled out by the practitioner using the AERF and collected by the researcher if occurring during the intervention. If participants contact the researcher after the intervention regarding an adverse event, then this will be logged by the researcher.

13. Participant timeline

See Table 3 for the participant timeline.

Table 3.
Participant timeline

		T_{-1}	T_0	T_1	T_2	T_3	F_1
Activity/ Assessment	Approx. time to complete	Pre-study Screening/ consent	Pre-study randomisation	Pre-intervention	Intervention	Post-intervention tests	Follow-up 1-week
Informed consent	5 mins	X					
Screening with DASS	5 mins	X					
Randomisation	15 mins		X				
Baseline assessment-questionnaires	15 mins			X			
Baseline assessment-physiological	15 mins			X			
Intervention	30 mins				X		
Post-intervention questionnaires	15 mins					X	
Post-intervention physiological	15 mins					X	
Telephone interview	30 mins						X

Abbreviations: T: Timepoint; F: Follow-up; DASS: Depression Anxiety Stress Scale.

14. Sample Size

For peer review only - http://bmjopen.bmj.com/site/about/guidelines.xhtml

The study will aim to recruit 32 participants. This number of participants is generally deemed sufficient for feasibility studies (49) and would represent approximately 10% of the sample size required in a full trial (50). This sample size also falls within what is practical given the available resources.

15. Recruitment

Recruitment at the university is being conducted by advertising in communal spaces with posters. Additionally, social media will be used for recruitment by reaching out to mental health support groups and sharing an advertisement for the study on various social networks (note: this recruitment work has begun). Participants will contact the research team if they are interested in taking part. They will then be given an information sheet to read and a consent form to sign. Following this they will complete the DASS to complete to determine their eligibility regarding mental health symptoms. The cut-off scores for mild to moderate will be defined as follows: depression = 10-20, anxiety = 8-14, and stress = 15-25 (51). If eligible they will be invited to take part in the intervention. If they display severe mental health symptoms, they will not be invited to take part further and signposted to the relevant mental health services and charities.

Methods: Assignment of interventions

16. Allocation

16.1 Sequence generation

The 32 participants will be randomly assigned to one of the four conditions using a computerised random number generator. Permuted block randomisation will be used to ensure that equal numbers of participants are in each condition. The block sizes will not be disclosed to help ensure concealment and prevent any potential prediction of group allocation. This will be conducted by the principal investigator (PI) of the study DJE, whilst the outcome assessor JH-B is blinded to this randomisation process.

For peer review only - http://bmjopen.bmj.com/site/about/guidelines.xhtml

16.2 Concealment mechanism

Allocation concealment will be ensured using sequentially numbered, sealed opaque envelopes which contain the group assignment. The PI will carry out the allocation concealment, and ensure that the outcome assessor is blinded to the intervention allocation.

16.3 Implementation

All participants who provide informed consent and who meet the eligibility criteria will be randomised into a study condition (as described in section 16.1). The randomiser DJE will not be directly involved in the recruitment or data collection, and instead, the outcome assessor will conduct the recruitment. The list of random numbers that correspond to group allocation will not be revealed to the researcher (JH-B) involved in data collection or recruitment. The sealed envelopes will contain a randomisation number and corresponding intervention identity code for the allocation of participants into the intervention groups. The osteopathic practitioner will then be able to open the envelope and determine which intervention is to be delivered on the day the study is conducted.

17.1 Blinding (masking)

The outcome assessor will be blind to the participant's group allocation. After pre-intervention psychometric and psychophysiological measures (see sections 12.3 and 12.4 respectively) have been completed, the outcome assessor will leave the room (to ensure blinding) and the intervention will begin, conducted by the osteopath. Participants will not be blinded to study intervention, as the osteopathic practitioner will need to explain study and intervention procedures, in line with the osteopathic practice standards and ethical consent (14). The practitioner will not be blinded to the intervention type (as they need to know what intervention to deliver) but will be blinded to the study outcomes. The outcome assessor will also be conducting the data analysis, and the random numbers corresponding to each group will only be revealed when this analysis has been completed. To ensure participants do not

For peer review only

disclose the condition they were allocated to, they will be asked not to communicate directly to the outcome assessor about the intervention they received. The study will therefore be single-blinded, where the outcome assessor is blind to intervention allocation, and the osteopathic practitioner will not be blind (hence single-blind).

17.2 Emergency unblinding

As the practitioner is not blinded, no emergency unblinding procedures are deemed necessary.

Methods: Data management and analysis

18. Data management

All data will be entered electronically at the university where the data is being collected and kept in a password-protected folder, which only the outcome assessor will have access to for the duration of the study. The electronic data will be kept confidential, and participants' names will not be linked to their datasets. For the longer term, electronic datasets will be kept indefinitely in the interest of transparency to fulfil any requests for the original data and maintained on the Open Science Framework (OSF).

19. Statistical Methods

19.1 Outcomes

Statistical analysis will be conducted using IBM SPSS (v. 27). Means and standard deviations will be reported for demographic data that includes gender, age, and ethnicity. For the main analysis, data will first be examined for normality using the Shapiro-Wilk test. If data is skewed, logarithmic transformation will be used, otherwise, analysis will continue without any transformation. HRV data will be pre-processed, and inspected for any potential artifacts, and these will be removed if identified. RMSSD will be calculated on the pre-processed artifact removed data using Kubios version 3.5[2] via Matlab version R2021a. Interoceptive

[2] https://www.kubios.com/

For peer review only - http://bmjopen.bmj.com/site/about/guidelines.xhtml

accuracy (IAc) will then be calculated using the formula: IAc = 1/4 Σ [1 − (|recorded heartbeats − counted heartbeats|/recorded heartbeats)]. The psychometrics will be totalled according to the relevant questionnaire instructions and subscales.

The main analysis will comprise of seven separate mixed design two (pre- and post-intervention) by four (AVHT, STM, CST, combination[3]) analysis of covariance (ANCOVA) models. This will comprise of five separate ANCOVAs for the five psychometrics (DASS, PANAS-SF, AAQ, SACS, MAIA) and another two ANCOVAs for the physiological measures of IAc and HRV (as measured by RMSSD and LF/HF ratio). Covariates will consist of (1) whether participants are currently receiving psychotherapy (yes or no) and (2) whether participants are receiving pharmacological treatment (yes or no). Significant models will be examined further using post hoc Bonferroni tests.

19.2 Additional Analyses

Exploratory correlational analyses will also be conducted to examine relationships between changes from pre- to post-intervention on the various measures (e.g., the relationship between change from pre-post HRV RMSSD and pre-post DASS scores).

19.3 Analysis of population and missing data

The study will operate on an intention-to-treat basis. All participants randomised and with pre-intervention data will be included in the final analysis. Any participants with missing data will be included in the analysis using the multiple imputation feature of SPSS.

Methods: Monitoring

20. Data monitoring

As this study is taking place over a short duration as a feasibility study and not a full RCT, no formal committee for data monitoring is required.

21. Harms

[3] Please see: Interventions section 11.1 for full details of these interventions.

For peer review only - http://bmjopen.bmj.com/site/about/guidelines.xhtml

The osteopathic practitioner will inform the participants about the general potential common adverse effects of osteopathy namely some stiffness and soreness in the days following the intervention, and rare adverse events including tissue damage (52), in line with informed consent processes. The osteopathic practitioner will record any adverse effects on the day the intervention is received (that occur during or immediately after the intervention) in the AERF (see Supplemental material 2). Participants will also be advised to contact the PI DJE by telephone if they have any concerns or adverse events following the intervention in subsequent days after the intervention was received. If such events are reported, these will again be reported by DJE in the AERF. Any adverse events or harms that are ranked highly on severity will be reported to the ethical committee. This includes any adverse events that for example require hospitalization.

22. Auditing

As the study is taking place over a short duration and only at one site, no formal auditing processes are deemed necessary, though PI will have regular team meetings to ensure the study is following the research protocol at all times.

Methods: Patient and public involvement statement

Key stakeholders were consulted and involved at a very early stage of the research process. The Patient Experience and Evaluation in Research (PEER)[4] group in the College of Human and Health Sciences at Swansea University were consulted. This group represented members of the public, students, and staff members, several of whom reported that they had experienced depression, anxiety, or stress at some point in their lives and emphasised the need for innovative approaches to the delivery of mental health support. The feasibility

[4] https://www.swansea.ac.uk/humanandhealthsciences/research-at-the-college-of-human-and-health/patientexperienceandevaluationinresearchpeergroup/)

For peer review only - http://bmjopen.bmj.com/site/about/guidelines.xhtml

design was explained to them, and they gave positive feedback about the nature of the preliminary research plan.

Ethics and dissemination

23. Research ethics approval

The protocol for this feasibility study has received ethical approval from the Department of Psychology Ethics Committee at Swansea University, ethical review reference number: 2022-5603-4810.

24. Protocol amendments

Any deviations from the protocol that could impact the conduct or bias of the study will be clearly outlined and justified in the final written report. Version control of the protocol using identifiers and dates, along with a list of amendments will be clearly listed. This will enable tracking of the history of amendments and identification of the most recent protocol version.

25. Consent

Participants will scan a QR code on recruitment posters or click a link via email/social media adverts that will take them to the study's information sheet. The information sheet emphasises that participation is voluntary and that they can withdraw from the study at any stage, without needing to provide a reason. If they have any questions or concerns at this stage, they are encouraged on the information sheet to contact the research team. If they are willing to proceed, they will complete an online consent form (see Supplemental material 3).

26. Ancillary research

The data collected in this study will not be used for any other ancillary research.

27. Confidentiality

Participants will be assigned a coded ID number to maintain confidentiality. Any records of personal identifiers such as informed consent forms will be stored separately from data with

For peer review only

ID numbers. To limit data access to the minimum number of individuals, only the researcher JHB will have access to the data for analysis.

28. Declaration of interests

The individual authors have no direct conflicts of interest to declare.

29. Access to data

Only the researcher JHB will have access to the dataset during the study period. Upon completion, the collected data will be deidentified and made available on the OSF. Similarly, the SPSS statistical syntax code used will be made available on OSF.

30. Ancillary and post-trial care

Participants will be fully debriefed once they have completed the study. The contact details of the research team will be provided should participants have any concerns. As the participants will be presenting with mild to moderate mental health symptoms, the debrief form will encourage participants to seek support services such as mental health charities or their GP if their psychological condition deteriorates at any time.

31. Dissemination policy

31.1 Trial results

Following the completion of the study, it is anticipated to take around 2-3 months to compile the final results ready for publication in an appropriate peer-reviewed journal. The study's results may also be used as part of presentations at any relevant conferences.

31.2 Authorship

The authors of this protocol will also be the authors of the final report. All authors have made substantive contributions to the design of the study. Additionally, all authors will have made substantive contributions to the interpretation of the data collected and the writing of the final report.

31.3 Reproducible research

For peer review only

This protocol will be available to researchers via open-access publication. The dataset collected will be deidentified and made available on OSF. Similarly, the statistical syntax code used will be made available on OSF. These will be made available no later than 1 year upon completion of data collection.

Acknowledgements

We would like to thank Stephen Hartshorn and James Bray for their contributions to delivering the osteopathic interventions.

Contributorship statement

JHB wrote the first draft, then assisted with subsequently revising additional drafts.

JHB wrote the first draft of this paper, then assisted with subsequently revising additional drafts. JHB also made substantive contributions to the concept, design, and writing of this study. JRD assisted with revising additional drafts of this paper, and also made substantive contributions to the concept, design, and writing of this study. DE was the principal investigator on the grant (Osteopathic Foundation) that funded this work. DJE therefore made substantial contributions to the design, concept, and writing of this study.

Competing interests

There are no competing interests. This project is funded by the Osteopathic Foundation (OF). The OF has no direct input into any aspects of this study. The individual authors have no direct conflicts of interest to declare.

Funding

This research has been funded by The Osteopathic Foundation, grant award number: URNLG010.

References

1. Chan AW, Tetzlaff JM, Gøtzsche PC, Altman DG, Mann H, Berlin JA, et al. SPIRIT 2013 explanation and elaboration: guidance for protocols of clinical trials. BMJ. 2013;346.
2. McManus S, Bebbington P, Jenkins R, Brugha T. Mental health and wellbeing in England: Adult Psychiatric Morbidity Survey 2014. Leeds; 2016.

For peer review only

3. WHO. Suicide worldwide in 2019: global health estimates. Geneva; 2021.
4. Edwards DJ. The textbook of health and social care. Washington, D.C: SAGE Publications Ltd; 2020.
5. Cipriani A, Furukawa TA, Salanti G, Chaimani A, Atkinson LZ, Ogawa Y, et al. Comparative efficacy and acceptability of 21 antidepressant drugs for the acute treatment of adults with major depressive disorder: a systematic review and network meta-analysis. The Lancet. 2018;391(10128):1357-66.
6. Munder T, Flückiger C, Leichsenring F, Abbass AA, Hilsenroth MJ, Luyten P, et al. Is psychotherapy effective? A re-analysis of treatments for depression. Epidemiology and Psychiatric Sciences. 2019;28(3):268-74.
7. Patel V, Maj M, Flisher AJ, De Silva MJ, Koschorke M, Prince M, et al. Reducing the treatment gap for mental disorders: a WPA survey. World Psychiatry. 2010;9(3):169-76.
8. Wang PS, Berglund PA, Olfson M, Kessler RC. Delays in Initial Treatment Contact after First Onset of a Mental Disorder. Health Services Research. 2004;39(2):393-416.
9. Edwards DJ, Kemp AH. A novel ACT-based video game to support mental health through embedded learning: a mixed-methods feasibility study protocol. BMJ Open. 2020;10(11):e041667-e.
10. Bohlen L, Shaw R, Cerritelli F, Esteves JE. Osteopathy and Mental Health: An Embodied, Predictive, and Interoceptive Framework. Frontiers in Psychology. 2021;12:4989-.
11. Edwards DJ, Toutt C. An evaluation of osteopathic treatment on psychological outcomes with patients suffering from chronic pain: A prospective observational cohort study collected through a health and well-being academy. Health psychology open. 2018;5(1).
12. Vaucher P, Macdonald RJD, Carnes D. The role of osteopathy in the Swiss primary health care system: a practice review. BMJ Open. 2021;8(8):e023770-e.
13. Council GO. General Osteopathic Council 2023 [Available from: https://www.osteopathy.org.uk/home/.
14. Council GO. Osteopathic Practice Standards London2019 [Available from: https://www.osteopathy.org.uk/standards/osteopathic-practice/.
15. Bagagiolo D, Rosa D, Borrelli F. Efficacy and safety of osteopathic manipulative treatment: an overview of systematic reviews. BMJ Open. 2022;12(4):e053468.
16. Rechberger V, Biberschick M, Porthun J. Effectiveness of an osteopathic treatment on the autonomic nervous system: a systematic review of the literature. European journal of medical research. 2019;24(1):1-14.
17. Zegarra-Parodi R, Park PYS, Heath DM, Makin IRS, Degenhardt BF, Roustit M. Assessment of skin blood flow following spinal manual therapy: A systematic review. Manual Therapy. 2015;20(2):228-49.
18. Kemp AH, Quintana DS, Felmingham KL, Matthews S, Jelinek HF. Depression, Comorbid Anxiety Disorders, and Heart Rate Variability in Physically Healthy, Unmedicated Patients: Implications for Cardiovascular Risk. PLOS ONE. 2012;7(2):e30777-e.
19. Pinna T, Edwards DJ. A Systematic Review of Associations Between Interoception, Vagal Tone, and Emotional Regulation: Potential Applications for Mental Health, Wellbeing, Psychological Flexibility, and Chronic Conditions. Frontiers in Psychology. 2020;11:1792-.
20. Cerritelli F, Cardone D, Pirino A, Merla A, Scoppa F. Does Osteopathic Manipulative Treatment Induce Autonomic Changes in Healthy Participants? A Thermal Imaging Study. Frontiers in neuroscience. 2020;14.
21. Arienti C, Farinola F, Ratti S, Daccò S, Fasulo L. Variations of HRV and skin conductance reveal the influence of CV4 and Rib Raising techniques on autonomic balance: A randomized controlled clinical trial. Journal of Bodywork and Movement Therapies. 2020;24(4):395-401.

For peer review only - http://bmjopen.bmj.com/site/about/guidelines.xhtml

22. Sherman KJ, Ludman EJ, Cook AJ, Hawkes RJ, Roy-Byrne PP, Bentley S, et al. Effectiveness of therapeutic massage for generalized anxiety disorder: a randomized controlled trial. Depression and anxiety. 2010;27(5):441-50.
23. Baumgart SBE, Baumbach Kraft A, Lorenz J. Effect of Psycho-Regulatory Massage Therapy on Pain and Depression in Women with Chronic and/or Somatoform Back Pain: A Randomized Controlled Trial. Brain sciences. 2020;10(10):1-13.
24. Sharpe PA, Williams HG, Granner ML, Hussey JR. A randomised study of the effects of massage therapy compared to guided relaxation on well-being and stress perception among older adults. Complementary Therapies in Medicine. 2007;15(3):157-63.
25. Castro-Sánchez AM, Aguilar-Ferrándiz ME, Matarán-Peñarrocha GA, Sánchez-Joya MDM, Arroyo-Morales M, Fernández-De-Las-Peñas C. Short-term effects of a manual therapy protocol on pain, physical function, quality of sleep, depressive symptoms, and pressure sensitivity in women and men with fibromyalgia syndrome: a randomized controlled trial. The Clinical journal of pain. 2014;30(7):589-97.
26. Espí-López GV, López-Bueno L, Vicente-Herrero MT, Martinez-Arnau FM, Monzani L. Efficacy of manual therapy on anxiety and depression in patients with tension-type headache. A randomized controlled clinical trial. International Journal of Osteopathic Medicine. 2016;22:11-20.
27. Cerritelli F, Cardone D, Pirino A, Merla A, Scoppa F. Does Osteopathic Manipulative Treatment Induce Autonomic Changes in Healthy Participants? A Thermal Imaging Study. Frontiers in Neuroscience. 2020;14:887-.
28. Cerritelli F, Chiacchiaretta P, Gambi F, Perrucci MG, Barassi G, Visciano C, et al. Effect of manual approaches with osteopathic modality on brain correlates of interoception: an fMRI study. Scientific Reports 2020 10:1. 2020;10(1):1-12.
29. Rapaport MH, Schettler P, Larson ER, Edwards SA, Dunlop BW, Rakofsky JJ, et al. Acute Swedish Massage Monotherapy Successfully Remediates Symptoms of Generalized Anxiety Disorder: A Proof-of-Concept, Randomized Controlled Study. The Journal of clinical psychiatry. 2016;77(7):e883-e91.
30. Seifert G, Kanitz JL, Rihs C, Krause I, Witt K, Voss A. Rhythmical massage improves autonomic nervous system function: a single-blind randomised controlled trial. Journal of integrative medicine. 2018;16(3):172-7.
31. Castro-Sánchez AM, Matarán-Peñarrocha GA, Sánchez-Labraca N, Quesada-Rubio JM, Granero-Molina J, Moreno-Lorenzo C. A randomized controlled trial investigating the effects of craniosacral therapy on pain and heart rate variability in fibromyalgia patients. Clinical rehabilitation. 2011;25(1):25-35.
32. Edwards DJ, Young H, Johnston R. The Immediate Effect of Therapeutic Touch and Deep Touch Pressure on Range of Motion, Interoceptive Accuracy and Heart Rate Variability: A Randomized Controlled Trial With Moderation Analysis. Frontiers in integrative neuroscience. 2018;12.
33. Creswell JW, Plano-Clark VL. Designing and Conducting Mixed Methods Research. 3rd ed. Los Angeles: SAGE Publishing; 2017.
34. Fryer G. Somatic dysfunction: An osteopathic conundrum. International Journal of Osteopathic Medicine. 2016;22:52-63.
35. Gabutti M, Draper-Rodi J. Osteopathic decapitation: Why do we consider the head differently from the rest of the body? New perspectives for an evidence-informed osteopathic approach to the head. International Journal of Osteopathic Medicine. 2014;17(4):256-62.
36. Rushton A, Carlesso LC, Flynn T, Hing WA, Rubinstein SM, Vogel S, et al. International Framework for Examination of the Cervical Region for Potential of Vascular Pathologies of the Neck Prior to Musculoskeletal Intervention: International IFOMPT Cervical Framework. Journal of Orthopaedic & Sports Physical Therapy. 2022;53(1):7-22.

For peer review only - http://bmjopen.bmj.com/site/about/guidelines.xhtml

37. Vaughan B, Moran R, Tehan P, Fryer G, Holmes M, Vogel S, et al. Manual therapy and cervical artery dysfunction: Identification of potential risk factors in clinical encounters. International Journal of Osteopathic Medicine. 2016;21:40-50.
38. Braun V, Clarke V. One size fits all? What counts as quality practice in (reflexive) thematic analysis? Qualitative Research in Psychology. 2021;18(3):328-52.
39. Lovibond PF, Lovibond SH. The structure of negative emotional states: Comparison of the Depression Anxiety Stress Scales (DASS) with the Beck Depression and Anxiety Inventories. Behaviour Research and Therapy. 1995;33(3):335-43.
40. Henry JD, Crawford JR. The short-form version of the Depression Anxiety Stress Scales (DASS-21): Construct validity and normative data in a large non-clinical sample. British Journal of Clinical Psychology. 2005;44(2):227-39.
41. Thompson ER. Development and Validation of an Internationally Reliable Short-Form of the Positive and Negative Affect Schedule (PANAS). Journal of Cross-Cultural Psychology. 2007;38(2):227-42.
42. Bond FW, Hayes SC, Baer RA, Carpenter KM, Guenole N, Orcutt HK, et al. Preliminary Psychometric Properties of the Acceptance and Action Questionnaire–II: A Revised Measure of Psychological Inflexibility and Experiential Avoidance. Behavior Therapy. 2011;42(4):676-88.
43. Zettle RD, Gird SR, Webster BK, Carrasquillo-Richardson N, Swails JA, Burdsal CA. The Self-as-Context Scale: Development and preliminary psychometric properties. Journal of Contextual Behavioral Science. 2018;10:64-74.
44. Mehling WE, Acree M, Stewart A, Silas J, Jones A. The Multidimensional Assessment of Interoceptive Awareness, Version 2 (MAIA-2). PLOS ONE. 2018;13(12):e0208034.
45. Reyes del Paso GA, Langewitz W, Mulder LJ, Van Roon A, Duschek S. The utility of low frequency heart rate variability as an index of sympathetic cardiac tone: a review with emphasis on a reanalysis of previous studies. Psychophysiology. 2013;50(5):477-87.
46. Schandry R. Heart Beat Perception and Emotional Experience. Psychophysiology. 1981;18(4):483-8.
47. Garfinkel SN, Seth AK, Barrett AB, Suzuki K, Critchley HD. Knowing your own heart: Distinguishing interoceptive accuracy from interoceptive awareness. Biological Psychology. 2015;104:65-74.
48. NICE. Hypertension in adults: diagnosis and management. London; 2022.
49. Lancaster GA, Dodd S, Williamson PR. Design and analysis of pilot studies: recommendations for good practice. Journal of evaluation in clinical practice. 2004;10(2):307-12.
50. Hertzog MA. Considerations in determining sample size for pilot studies. Research in Nursing & Health. 2008;31(2):180-91.
51. Lovibond SH, Lovibond PF. Manual for the Depression Anxiety Stress Scales. 2nd ed. Sydney: Psychology Foundation; 1995.
52. Carnes D, Mars TS, Mullinger B, Froud R, Underwood M. Adverse events and manual therapy: A systematic review. Manual Therapy. 2010;15(4):355-63.

For peer review only

SPIRIT

STANDARD PROTOCOL ITEMS: RECOMMENDATIONS FOR INTERVENTIONAL TRIALS

SPIRIT 2013 Checklist: Recommended items to address in a clinical trial protocol and related documents*

Section/item	Item No	Description	Addressed on page number
**Administrative information**			
Title	1	Descriptive title identifying the study design, population, interventions, and, if applicable, trial acronym	Page 3, Section 1.
Trial registration	2a	Trial identifier and registry name. If not yet registered, name of intended registry	Page 3, Section 2.
	2b	All items from the World Health Organization Trial Registration Data Set	N/A
Protocol version	3	Date and version identifier	Page 3, Section 3.
Funding	4	Sources and types of financial, material, and other support	Page 3, Section 4.
Roles and responsibilities	5a	Names, affiliations, and roles of protocol contributors	Pages 3-4, Section 5.1
	5b	Name and contact information for the trial sponsor	Page 4, Section 5.2
	5c	Role of study sponsor and funders, if any, in study design; collection, management, analysis, and interpretation of data; writing of the report; and the decision to submit the report for publication, including whether they will have ultimate authority over any of these activities	Page 4, Section 5.3

For peer review only

	5d	Composition, roles, and responsibilities of the coordinating centre, steering committee, endpoint adjudication committee, data management team, and other individuals or groups overseeing the trial, if applicable (see Item 21a for data monitoring committee)	N/A
**Introduction**			
Background and rationale	6a	Description of research question and justification for undertaking the trial, including summary of relevant studies (published and unpublished) examining benefits and harms for each intervention	Pages 4-5, Section 6.1
	6b	Explanation for choice of comparators	Pages 5-7, Section 6.2
Objectives	7	Specific objectives or hypotheses	Page 7, Section 7
Trial design	8	Description of trial design including type of trial (eg, parallel group, crossover, factorial, single group), allocation ratio, and framework (eg, superiority, equivalence, noninferiority, exploratory)	Page 7, Section 8
**Methods: Participants, interventions, and outcomes**			
Study setting	9	Description of study settings (eg, community clinic, academic hospital) and list of countries where data will be collected. Reference to where list of study sites can be obtained	Pages 7-8, Section 9
Eligibility criteria	10	Inclusion and exclusion criteria for participants. If applicable, eligibility criteria for study centres and individuals who will perform the interventions (eg, surgeons, psychotherapists)	Page 8, Section 10
Interventions	11a	Interventions for each group with sufficient detail to allow replication, including how and when they will be administered	Pages 8-11, Section 11.1
	11b	Criteria for discontinuing or modifying allocated interventions for a given trial participant (eg, drug dose change in response to harms, participant request, or improving/worsening disease)	Page 11, Section 11.2
	11c	Strategies to improve adherence to intervention protocols, and any procedures for monitoring adherence (eg, drug tablet return, laboratory tests)	Page 11-12, Section 11.3

For peer review only

	11d	Relevant concomitant care and interventions that are permitted or prohibited during the trial	Page 12, Section 11.4
Outcomes	12	Primary, secondary, and other outcomes, including the specific measurement variable (eg, systolic blood pressure), analysis metric (eg, change from baseline, final value, time to event), method of aggregation (eg, median, proportion), and time point for each outcome. Explanation of the clinical relevance of chosen efficacy and harm outcomes is strongly recommended	Pages 12-18, Section 12
Participant timeline	13	Time schedule of enrolment, interventions (including any run-ins and washouts), assessments, and visits for participants. A schematic diagram is highly recommended (see Figure)	Pages 18-19, Section 13 (Table 3)
Sample size	14	Estimated number of participants needed to achieve study objectives and how it was determined, including clinical and statistical assumptions supporting any sample size calculations	Page 19, Section 14
Recruitment	15	Strategies for achieving adequate participant enrolment to reach target sample size	Page 19, Section 15.

Methods: Assignment of interventions (for controlled trials)

Allocation:

Sequence generation	16a	Method of generating the allocation sequence (eg, computer-generated random numbers), and list of any factors for stratification. To reduce predictability of a random sequence, details of any planned restriction (eg, blocking) should be provided in a separate document that is unavailable to those who enrol participants or assign interventions	Page 20, Section 16.1
Allocation concealment mechanism	16b	Mechanism of implementing the allocation sequence (eg, central telephone; sequentially numbered, opaque, sealed envelopes), describing any steps to conceal the sequence until interventions are assigned	Page 20, Section 16.2
Implementation	16c	Who will generate the allocation sequence, who will enrol participants, and who will assign participants to interventions	Page 20, Section 16.3
Blinding (masking)	17a	Who will be blinded after assignment to interventions (eg, trial participants, care providers, outcome assessors, data analysts), and how	Pages 20-21, Section 17.1

	17b	If blinded, circumstances under which unblinding is permissible, and procedure for revealing a participant's allocated intervention during the trial	Page 21, Section 17.2.
**Methods: Data collection, management, and analysis**			
Data collection methods	18a	Plans for assessment and collection of outcome, baseline, and other trial data, including any related processes to promote data quality (eg, duplicate measurements, training of assessors) and a description of study instruments (eg, questionnaires, laboratory tests) along with their reliability and validity, if known. Reference to where data collection forms can be found, if not in the protocol	Pages 12-18, Section 12.
	18b	Plans to promote participant retention and complete follow-up, including list of any outcome data to be collected for participants who discontinue or deviate from intervention protocols	N/A as data only collected pre-post.
Data management	19	Plans for data entry, coding, security, and storage, including any related processes to promote data quality (eg, double data entry; range checks for data values). Reference to where details of data management procedures can be found, if not in the protocol	Page 21, Section 18.
Statistical methods	20a	Statistical methods for analysing primary and secondary outcomes. Reference to where other details of the statistical analysis plan can be found, if not in the protocol	Page 21-22, Section 19.1
	20b	Methods for any additional analyses (eg, subgroup and adjusted analyses)	Page 22, Section 19.2
	20c	Definition of analysis population relating to protocol non-adherence (eg, as randomised analysis), and any statistical methods to handle missing data (eg, multiple imputation)	Page 22, Section 19.3
**Methods: Monitoring**			
Data monitoring	21a	Composition of data monitoring committee (DMC); summary of its role and reporting structure; statement of whether it is independent from the sponsor and competing interests; and reference to where further details about its charter can be found, if not in the protocol. Alternatively, an explanation of why a DMC is not needed	Page 23, Section 20.
	21b	Description of any interim analyses and stopping guidelines, including who will have access to these interim results and make the final decision to terminate the trial	N/A

Harms	22	Plans for collecting, assessing, reporting, and managing solicited and spontaneously reported adverse events and other unintended effects of trial interventions or trial conduct	Page 23, Section 21.
Auditing	23	Frequency and procedures for auditing trial conduct, if any, and whether the process will be independent from investigators and the sponsor	Page 23, Section 22.
**Ethics and dissemination**			
Research ethics approval	24	Plans for seeking research ethics committee/institutional review board (REC/IRB) approval	Page 24, Section 23.
Protocol amendments	25	Plans for communicating important protocol modifications (eg, changes to eligibility criteria, outcomes, analyses) to relevant parties (eg, investigators, REC/IRBs, trial participants, trial registries, journals, regulators)	Page 24, Section 24
Consent or assent	26a	Who will obtain informed consent or assent from potential trial participants or authorised surrogates, and how (see Item 32)	Page 24, Section 25
	26b	Additional consent provisions for collection and use of participant data and biological specimens in ancillary studies, if applicable	N/A
Confidentiality	27	How personal information about potential and enrolled participants will be collected, shared, and maintained in order to protect confidentiality before, during, and after the trial	Pages 24-25, Section 27.
Declaration of interests	28	Financial and other competing interests for principal investigators for the overall trial and each study site	Page 25, Section 28.
Access to data	29	Statement of who will have access to the final trial dataset, and disclosure of contractual agreements that limit such access for investigators	Page 25, Section 29.
Ancillary and post-trial care	30	Provisions, if any, for ancillary and post-trial care, and for compensation to those who suffer harm from trial participation	Page 25, Section 30.
Dissemination policy	31a	Plans for investigators and sponsor to communicate trial results to participants, healthcare professionals, the public, and other relevant groups (eg, via publication, reporting in results databases, or other data sharing arrangements), including any publication restrictions	Page 25, Section 31.1

For peer review only

	31b	Authorship eligibility guidelines and any intended use of professional writers	Page 25, Section 31.2
	31c	Plans, if any, for granting public access to the full protocol, participant-level dataset, and statistical code	Page 26, Section 31.3
**Appendices**			
Informed consent materials	32	Model consent form and other related documentation given to participants and authorised surrogates	Supplemental material 2
Biological specimens	33	Plans for collection, laboratory evaluation, and storage of biological specimens for genetic or molecular analysis in the current trial and for future use in ancillary studies, if applicable	N/A

*It is strongly recommended that this checklist be read in conjunction with the SPIRIT 2013 Explanation & Elaboration for important clarification on the items. Amendments to the protocol should be tracked and dated. The SPIRIT checklist is copyrighted by the SPIRIT Group under the Creative Commons "Attribution-NonCommercial-NoDerivs 3.0 Unported" license.

For peer review only

Supplemental material 1.
Adverse events report form

Adverse Events Report Form		
**Practitioner ID:**		**Patient ID:**
**Date:**		**Location:**
**Description of adverse event:**		
**Actions taken:**<br>What?<br>When?<br>By whom?<br>Outcome?		
**Further actions needed?**		

For peer review only

Supplemental material 2.

Consent form

Participant Consent Form

Project title: **Measuring psychophysiological outcomes in a therapeutic touch approach.**

You must be age 18 or over to complete this study.

Name and Contact details of the principal researchers: Dr Josh Hope-Bell j.b.hope-bell@swansea.ac.uk and Dr Darren Edwards d.j.edwards@swansea.ac.uk.

This study is being conducted by Swansea University, Faculty of Medicine, Health and Life Sciences.

- I (the participant) consent to participate in the study
- I confirm that I have read and understand the information provided in relation to this study.
- I understand that my participation is voluntary. I understand that I am free to withdraw at any time during the study but once I have completed all phases of the study, withdrawal will not be possible because data will be completely anonymised.
- I understand what my role will be in this research, and all my questions have been answered to my satisfaction.
- I have been informed that the information I provide will be safeguarded.
- I am happy for the information I provide to be used (anonymously) in academic papers and other formal research outputs, however my name will not be published so anonymity is ensured.
- I agree to the researchers processing my personal data in accordance with the aims of the study described in the participant information.
- I am age 18 years or above.

If you agree with all statements above, click **Yes (I consent)**

If you disagree with any of the statements above, click **No (I do not consent)**

For peer review only
